# Investigating Foraging Niches for Tenrecs, Bats, and Rodents at Betampona Réserve Naturelle Intégrale (Central Eastern Madagascar) Using Stable Carbon and Nitrogen Isotopes in Fur and Bone

**DOI:** 10.3390/ani15162423

**Published:** 2025-08-19

**Authors:** Brooke Erin Crowley, Steven Michael Goodman

**Affiliations:** 1 Department of Geosciences, University of Cincinnati, 500 Geology Physics Building, Cincinnati, OH 45221-0013, USA; 2 Department of Anthropology, University of Cincinnati, 481 Braunstein Hall, Cincinnati, OH 45221-0380, USA; 3 Negaunee Integrative Research Center, Field Museum of Natural History, 1400 South DuSable Shore Drive, Chicago, IL 60605, USA; sgoodman@fieldmuseum.org; 4 Association Vahatra, BP 3972, Antananarivo 101, Madagascar

**Keywords:** keratin, collagen, edge effects, Chiroptera, Muridae, Nesomyinae, Soricidae, Tenrecidae

## Abstract

We used stable carbon and nitrogen isotope values in plants, fur, and bone to establish baseline isotopic variability and investigate food web dynamics for small mammals at Betampona Réserve Naturelle Intégrale (BRNI), which protects an isolated parcel of tropical lowland moist evergreen forest in central eastern Madagascar. We included foliage from four understory plant genera, 13 endemic mammal species (5 bats, 5 tenrecs, and 3 nesomyine rodents), and introduced *Rattus rattus* and *Suncus murinus* that were collected along a gradient of degraded to intact forest from the southern boundary to the interior of BRNI as well as nearby villages. Isotopic data indicate niche partitioning among mammal groups, as well as among species within each of the groups. Most species appear to have foraged in the forest understory, although some, bats in particular, may have foraged outside of the protected area. We did not find evidence for competition between introduced and endemic animals but caution that some sample sizes are small. These results provide previously unknown insight into dietary preferences for some of Madagascar’s least studied mammals and establish a foundation for future work at BRNI.

## Simple Summary

Madagascar is an island with incredible biodiversity, much of which occurs nowhere else on Earth (endemic). Yet there is a lot we still do not know about the biology of Madagascar’s wildlife. Establishing what the island’s species eat and where they forage is critical to their long-term protection. We used chemistry to indirectly study diet for several small mammal species that are native or endemic to Madagascar, including bats, rodents, and a unique group of animals called tenrecs, living in or near a forested protected area in central eastern Madagascar. We also investigated possible competition for resources with introduced rats and shrews. We found evidence for most species consuming different resources and also some surprising results that may reflect geographic or seasonal dietary variability. These data provide an initial picture of foraging behavior and competition for poorly understood species and contribute to the growing literature from elsewhere on Madagascar that will ultimately help us better understand the natural history and behavior of animals that are hard to observe and can be used to guide management decisions.

## 1. Introduction

Madagascar, the world’s fourth largest island, is a global biodiversity hotspot, boasting an incredible array of plants, arthropods, and vertebrates, reviewed in [1]. Unfortunately, the island has experienced numerous recent extinctions, reviewed in [2], and many of its remaining endemic animals and plants are threatened with extinction, reviewed in [3]. Understanding and protecting the island’s biodiversity (as well as the ecosystem services it provides) have, therefore, been repeatedly identified as a conservation priority, e.g., [3,4,5,6,7,8].

Madagascar’s primates (lemurs) have received considerable research and conservation attention over the past few decades, reviewed in [9]. There are now five editions of *Lemurs of Madagascar* by Russell Mittermeier and colleagues, and the most recent, published in 2023 [10], is nearly 1000 pages long. Yet comparatively little work has been conducted on other endemic mammalian lineages, such as afrosoricid tenrecs (family Tenrecidae), Malagasy rodents (family Nesomyidae, subfamily Nesomyinae), or the island’s diverse bats. Species within these non-primate groups tend to be small, nocturnal, and forage individually, which makes it challenging to directly observe them. Consequently, there are many gaps in our understanding of their natural history, including feeding behavior, how they interact with each other, and how they are impacted by both direct and indirect human activities, such as land use, extraction of forest products, bush meat consumption, and presence of introduced animals (which can predate and compete with native taxa and also transmit diseases). If we want to protect remaining endemic species and ensure they have a future, we need to know more about their ecological requirements.

Stable carbon (δ^13^C) and nitrogen (δ^15^N) isotope values can provide a means of indirectly monitoring foraging behavior, niche partitioning, and potential competition among co-occurring animals, reviewed in [11]. Stable isotopes have been successfully (and increasingly) used to study the dietary and habitat preferences of both native and introduced animals on Madagascar. A recent compilation of modern isotope data for the island (called IsoMad) includes nearly 20,000 data points [12]. Researchers can now use this publicly available dataset to investigate niche partitioning and food web dynamics for various vertebrates within and among different bioclimatic regions on the island. Nevertheless, despite its impressive size, the database is currently somewhat limited in the taxa, habitats, and geographic regions it includes. For example, lemurs and introduced murid rats are well represented, but very few data exist for other endemic vertebrates.

**Figure 1:**
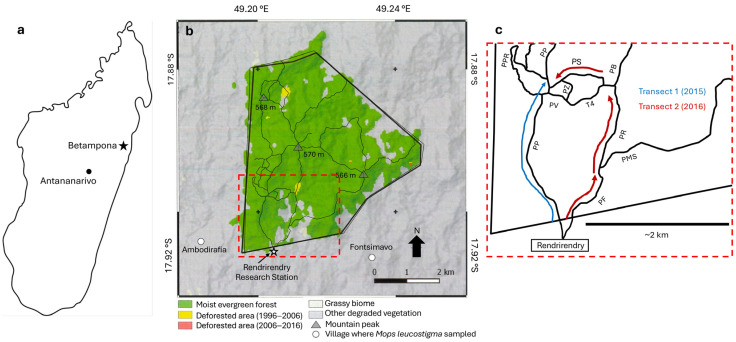
Maps showing: (**a**) the location of Betampona Réserve Naturelle Intégrale (BRNI) and the capital city, Antananarivo, in Madagascar; (**b**) topography and vegetation cover at BRNI, as well as locations of nearby villages where *Mops leucostigma* were captured (Fontsimavo 17°56′ S, 49°14′ E, and Ambodirafia 17°55.677′ S, 49°10.896′ E); and (**c**) the trail system and directionality of transects in southwestern BRNI where this study took place. Trail map adapted from a GIS shapefile provided by the Madagascar Fauna and Flora Group (https://www.madagascarfaunaflora.org/betampona-natural-reserve.html, accessed on 1 May 2025). BRNI base map adapted from [15]; “+” symbols are included for latitude and longitude orientation. There are some subtle differences in the protected area boundary between the official legislation naming the site in decree no. 66-242 of 1 June 1966 (lighter line) and the official shape files dating from 2017 of the Direction du Système des Aires Protégées (heavier line).

We undertook an initial isotopic survey of Betampona Réserve Naturelle Intégrale (BRNI) in central eastern Madagascar (Figure 1). This reserve is primarily lowland moist evergreen forest, sensu [13], which is a habitat that has been targeted as a conservation priority [14] and is not well represented in the current IsoMad database. We present new δ^13^C and δ^15^N data for foliage from four understory plant genera (*Dypsis*, *Ravenala*, *Pandanus*, and *Psidium*), as well as fur and bone from multiple small mammal taxa that are underrepresented in the ISOMAD database: five bats (*Macronycteris commersoni*, *Myotis goudoti*, *Rousettus madagascariensis*, *Myzopoda aurita*, and *Mops leucostigma*); five tenrecs (*Hemicentetes semispinosus*, *Setifer setosus*, *Nesogale dobsoni*, *Nesogale talazaci*, and *Oryzorictes hova*); three nesomyine rodents (*Eliurus minor*, *Eliurus petteri*, and *Eliurus webbi*); and two introduced small mammals, a soricid shrew (*Suncus murinus*) and murid rat (*Rattus rattus*).

We use foliar δ^13^C and δ^15^N values to establish baseline spatial isotopic variability for southern BRNI and then explore isotopic niches for the various small mammal taxa. The sample size is small for several species. Nevertheless, isotopic data provide evidence for these species’ diet and habitat preferences at BRNI, corroborating (or modifying) what we think we know about them (Table 1). While we consider this study to be somewhat exploratory due to limited sample sizes, the data we present can act as a starting point for additional isotopic research at BRNI and can also serve as a comparative reference for monitoring the foraging ecology of small mammals elsewhere in eastern Madagascar, including disturbed habitats. We have added these new data to the IsoMad database so they are publicly available (https://pandoradata.earth/dataset/isomad-modern-biological-material, accessed on 1 May 2025).

### 1.1. Site Description

BRNI was established in 1927 and is among Madagascar’s first set of protected areas. It encompasses 2228 hectares of lowland moist evergreen forest in eastern Madagascar (Figure 1) and is one of the last remaining large parcels of relatively undisturbed lowland forest in the central eastern portion of the island [15,34,35]. Since 2015, BRNI has been included in the Ankenihemy-Zahamena corridor along with three Parc National (Zahemana, Analamazaotra, and Mantadia), and one other Réserve Spéciale (Mangerivola). This corridor encompasses 371,000 hectares of forest, reviewed in [36]. However, BRNI sits at lower elevations than the other protected areas and is physically separated from them by ≥15 km of degraded forest and agricultural areas.

BRNI has received a high ranking with reference to forested areas on western Indian Ocean islands for its importance in conserving the unique biodiversity of Madagascar [14]. Despite its relatively small size, BRNI is notably species-rich (both in terms of plants and animals). This is likely a result of certain groups being represented by local microendemism (e.g., a number of frog species seem to be restricted to the site [37,38,39]), as well as a reflection of the regional climate, which is hot and moist. Between 2004 and 2013, average annual minimum and maximum temperatures at BRNI’s research station (Rendrirendry) ranged from 15.2 to 39.1 °C, and annual rainfall was 3130.4 mm (https://www.madagascarfaunaflora.org/betampona-natural-reserve.html, accessed on 1 May 2025). More rain falls between November and April (ca. 64%), but there is no distinct dry season. Additionally, although it is surrounded by anthropogenic vegetation, BRNI has not suffered in any significant manner from human impacts within its boundaries [34]. This is primarily due to BRNI’s status as a reserve naturelle intégrale or strict nature reserve; tourism is prohibited, and access is only available to researchers with permits [40]. Finally, biodiversity is likely bolstered by BRNI’s topographical complexity. The reserve is characterized by steep slopes and deep river valleys (Figure 1); the vast majority of the reserve (ca. 1926 hectares) is between 251 and 500 m above sea level (masl), but there are several valleys <251 masl, and several peaks are taller than 500 masl [15]. Typical vegetation at BRNI has multiple layers, with emergent trees as tall as 40 m from the forest floor, a dense upper canopy that is 20–30 m high, a subcanopy tree layer between 8 and 16 m, and a shrub layer [34,35]. Ridges tend to have fewer large trees than more protected valleys [34]. Approximately 20% of BRNI canopy cover has gaps (some >10 m) created by forceful storms, including infrequent cyclones and associated landslides. Vegetation in these regenerating areas is dominated by smaller trees (1–5 cm in diameter) or dense clumps of colonizing species, including bamboo, traveler’s palm (*Ravenala* spp., family Strelitziaceae), a variety of true palms (Arecaceae), and pantropical ginger (*Aframomum angustifolium*, family Zingiberaceae), and guava (*Psidium cattleianum*, family Myrtaceae) [34,35].

### 1.2. Mammal Species Overview

As summarized in Table 1, tenrecs, at least the species we analyzed, are thought to primarily forage on the forest floor in leaf litter and shallow burrow systems. Their diet is understood to be dominated by arthropods, especially annelid earthworms, but some species also eat mollusks, small vertebrates, eggs, and sometimes fruit. Bats primarily forage on volant insects (which are almost certainly distinct from the prey targeted by tenrecs), but some may also be able to glean non-volant arthropods off of vegetation [41,42,43]. *Rousettus madagascariensis* is a fruit bat and, therefore, should consume minimal animal matter. Endemic nesomyine rodents are also thought to be primarily vegetarian and eat a mix of fruits and seeds but may also consume some arthropods. *Eliurus* species may also differ in their degree of terrestrial versus arboreal habit. However, dietary information for most of the analyzed species is sparse (and mostly informed by fecal or stomach content analysis), and habit inferences are based on where animals have been trapped (which in turn is biased by trap location, typically between 0 and 3 m from the forest floor). Many of the small mammals at BRNI have not been directly observed in the wild, and it is possible that indirect inferences about their behavior (e.g., from stomach content analysis) are not accurate. Introduced shrews should have diets similar to tenrecs (primarily comprising arthropods and even small mammals, including house mice), but they are also known to eat grains and human foods when available. They tend to be terrestrial but may clamber over rocks and logs. Finally, murid rats tend to be somewhat arboreal and highly omnivorous; they will readily consume eggs and small vertebrates like baby rodents and birds if given the opportunity.

### 1.3. Background on Carbon and Nitrogen Isotopes

A plant’s photosynthetic pathway is the primary factor influencing its δ^13^C values. Trees, shrubs, and herbs typically use C_3_ photosynthesis, while most tropical grasses use C_4_ photosynthesis, reviewed in [44]. C_4_ plants can only thrive in relatively warm and sunny habitats and typically have relatively consistent δ^13^C values between ca. −12 and −15‰, reviewed in [45]. In contrast, C_3_ plants can have highly variable δ^13^C values ranging from ca. −20 to −37‰, reviewed in [46]. This variability is primarily driven by temperature and moisture availability. Plants that grow in drier habitats or experience higher temperatures have higher δ^13^C values than those in moister habitats or experience lower temperatures, reviewed in [46]. However, there can be pronounced isotopic variability even among microhabitats at the same locality. For example, C_3_ plants growing near the forest floor under a dense tree canopy tend to have very low δ^13^C values, while canopy vegetation at the same locality has higher δ^13^C values, and understory plants growing under a less continuous canopy, in gaps, or near forest edges have intermediate δ^13^C values [47,48]. These patterns, referred to as canopy and edge effects, reflect differences in relative humidity and the amount of light plants receive among different microhabitats, as well as the uptake of CO_2_ respired by soil microorganisms close to the forest floor, reviewed in [49].

Nitrogen isotopes in vegetation primarily reflect how a plant obtains nitrogen. Plants that are able to access atmospheric N_2_ via symbiotic nitrogen-fixing bacteria (e.g., most legumes) have δ^15^N values close to the atmospheric value (0‰), reviewed in [50]. In contrast, plants that take up nitrate or ammonium from the soil have variable δ^15^N values depending on the isotopic composition of the nitrogen source, the degree to which they rely on symbiotic fungal mycorrhizae (as well as the type of mycorrhizal association), and growing conditions, reviewed in [50,51,52]. Like carbon isotopes, plant δ^15^N values are influenced by temperature and moisture availability, with higher values being observed for plants in dry, hot habitats, reviewed in [52]. Plants growing in the relatively moist environment at BRNI would be expected to have low δ^15^N values (likely <0‰) [12,52]. Plant δ^15^N values are less influenced by the canopy effect, but there may still be measurable differences in δ^15^N values between understory and canopy vegetation, which likely reflect differences in leaf size, rooting depth, species composition, and potentially the incorporation of atmospherically derived N by canopy foliage [47,53,54].

One might also expect δ^15^N values to increase from a forest interior towards a forest edge due to higher temperatures and lower relative humidity and soil moisture. However, to our knowledge, only one study has evaluated this explicitly. Crowley and colleagues [55] explored edge effects for foliar δ^13^C and δ^15^N in a dry forest of northwestern Madagascar and, unexpectedly, observed the opposite trend; foliar δ^15^N values increased with increasing distance from the forest edge. They considered several possible explanations, including anthropogenic influence, variable organic matter input, tree rooting depth, and soil type. None of these potential variables were entirely satisfactory, and the authors ultimately concluded that differences in soil type may have resulted in spatial differences in plant available N and possibly lower soil moisture content in the forest interior. Topography, which is tied to microclimate conditions, soil depth and moisture availability, as well as nutrient cycling, can also have a considerable influence on both foliar δ^13^C and δ^15^N values [56,57,58,59,60,61]. Finally, there may be small differences in δ^13^C and δ^15^N values between different types of plant tissues, such as leaves, fruits, flowers, seeds, and roots [62,63,64,65,66], although we would expect these to be small (ca. 1–2‰).

Carbon and nitrogen isotope values in mammalian fur keratin or bone collagen reflect both consumed foods (diet) and where an animal prefers to forage (habitat). Isotope values in an herbivore’s organic tissues reflect those in consumed plants, with some offset. We would expect proteinaceous tissues for herbivorous, small-bodied mammals to have δ^13^C and δ^15^N values that are, respectively, ca. 3‰ and 5‰ higher than those in the foods they consume [67,68]. More faunivorous animals (such as omnivorous tenrecs and insectivorous bats) have higher δ^15^N and potentially higher δ^13^C values than strict herbivores, reviewed in [11,69,70]. There may be isotopic differences between bone collagen and fur keratin from the same individual, but these should be small (<1‰; reviewed in [71,72,73]). Additionally, animals that forage on the forest floor should be isotopically distinguishable from those that forage in the canopy at the same site or routinely outside of the forest, especially if they consume C_4_ foods [47,74,75]. Finally, there can be small isotopic differences among co-occurring folivores, frugivores, and granivores, although these differences may be an artifact of the microhabitat in which the different animals foraged rather than diet per se [47,76,77].

## 2. Methods

### 2.1. Sample Collection

We collected mature leaves from four frequently encountered understory tree genera: *Dypsis* (Arecaceae), *Pandanus* (Pandanaceae), *Ravenala* (Strelitziaceae), and *Psidium* (Myrtaceae). These taxa tend to be pioneers that colonize disturbed areas. There are at least eight species of *Dypsis* at BRNI, several of which are locally endemic to central eastern lowland Madagascar [78]. There are also several locally occurring species of *Pandanus* at BRNI [79]. Until recently, only one species of *Ravenala* was recognized. However, Haevermans and colleagues [80] described five new species in 2021. Based on the distribution of members of this genus in central eastern Madagascar, at least two species could be present at BRNI.

Leaves were collected approximately every 200 m along two transects that followed established trail systems with measured meter markers in southern BRNI (Figure 1). Each transect started at the southern edge of BRNI and continued several kilometers into the forest interior. The first transect (T1), which was sampled at the end of the dry season in November 2015, followed “Piste Principale” (PP) along a ridgeline for ca. 3000 m. For the first 800 m of the transect, vegetation was disturbed and the forest dominated by *Psidium* and *Ravenala*, while the remaining 2200 m was largely intact forest. We sampled *Ravenala* along the entirety of T1, but *Psidium* was only present for the first ca. 800 m of the trail, while *Dypsis* and *Pandanus* were present along the remainder of the transect. The second transect (T2), which was sampled in December 2016 (at the start of the rainy season), followed “Piste Fontsimavo” (PF) for ca. 1500 m into the forest interior, then “Piste Ranomena” (PR) for 800 m, and finally “Piste Sahabefoza” (PS) for ca. 1000 m, terminating very close to where T1 ended (Figure 1). This transect covered more variable topography, including ridges, slopes, and valleys. All of PF, and the initial 200 m of PR, ran through heavily disturbed forest and open areas, while forest vegetation along the last 400 m of PR and all of PS was intact or largely intact. No *Psidium* was collected along T2. *Dypsis* and *Ravenala* were collected along the entire transect, and *Pandanus* was sampled ≥400 m from the transect start point. All foliage samples from both transects were collected ca. 1–2.5 m above the forest floor and were dried in sealable baggies filled with desiccating beads. Details for each sample are provided in Supplementary Material Table S1.

As part of a biological survey of the non-primate mammals at BRNI, SMG and colleagues trapped a variety of native or endemic bats, tenrecs, and rodents, as well as three introduced murid *Rattus rattus* individuals and one Asian soricid shrew (*Suncus murinus*). This survey was conducted during the same sampling periods as T1 and T2 (November 2015 and December 2016, respectively) and along the same major trail systems in southern BRNI (Figure 1; Table 1) or just beyond the reserve’s boundaries, in the case of some bats.

Small mammals were trapped using live Sherman or National traps (mostly for rodents), pitfall traps (mostly for tenrecs), or mist or harp nets (for bats). Most Sherman and National traps were placed in a variety of settings on the ground, while ca. 20% were in arboreal positions no more than 2 m from the forest floor. Traps were baited with unsalted peanut butter, which was renewed daily in the late afternoon, and checked twice per day for captured animals, just after dawn and in the late afternoon. For further details on field trapping techniques, see [81].

Bats were trapped using either Japanese mist nets (6 or 12 m long × 2.6 m tall, with four pockets and 24 or 36 mm mesh) placed across flight pathways or two-bank Faunatech Austbat harp traps installed along trails that might serve as bat flyways, often in areas where constrictions in vegetation formed narrow passages. Further, harp traps were placed in front of exit points from bat roosting sites in human structures. These devices were installed before sunset and disassembled after dawn; in some cases, mist nets were closed from around 11 pm to 3 am, which was a period of notably reduced bat activity.

All sampled individuals were designated as adult or subadult based on different external morphological characters and the state of reproductive organs. Summary information about each species captured is provided in Table 1; for detailed information related to each specimen, see Table S2. Collected animals were euthanized, and a fur or spine sample was collected; conversely, the entire limb (tail, wing, leg) was amputated after death for isotopic analysis. Samples were stored in 70% ethanol. Specimens were deposited with the Field Museum of Natural History (FMNH), in Chicago, USA, or the Université d’Antananarivo Département de Biologie Animale (and UADBA) in Madagascar.

Specimens were collected in accordance with the Institut Pasteur (Paris) guidelines for animal husbandry and experiments (www.pasteur.fr/ip/easysite/pasteur/en/institut-pasteur/ethics-charter, accessed on 1 May 2025). No national committee for animal welfare existed on Madagascar during the period of this study, so the protocol was approved and validated by the Ad hoc CAFF/CORE committee in Madagascar. All biological material was collected with permits from the Malagasy authorities, specifically the Minister de l’Environnement et Développement Durable, Direction du Systeme des Aires Protégées (permit numbers 259/15/MEEMF/SG/DGF/DAPT/SCBT of 12 October 2015 and 283/16/MEEMF/SG/DGF/DSAP/SCB.Re of 1 December 2016) and export permits from the same ministry (160N-EA05/MG16 of 5 May 2016 and 056N-EA03/MG17 of 7 March 2017).

### 2.2. Sample Preparation

Samples were shipped to Cincinnati, OH, USA, for analysis and processed in BEC’s Quaternary Paleoecology Laboratory at the University of Cincinnati (UC). Approximately 200 mg of each dried plant sample was flash frozen with liquid nitrogen and homogenized using an agate mortar and pestle. Small mammal tissue samples were removed from ethanol and dried upon arrival. When available, fur, hair, or spines were removed from amputated limbs and cleaned by soaking in methanol and air drying. Bones from small mammals were defleshed by soaking in a mild solution of Tide^®^ Free and Gentle Detergent and Adolph’s Meat Tenderizer at <60 °C in a slow cooker, following [75,82], scrubbing with a toothbrush, rinsing with ultrapure water and air drying. Bone was demineralized following Crowley et al. [73]. Approximately 50 mg of small elements or fragments was soaked in 0.5 N HCl at 4 °C until samples were pliable. Acid was refreshed as needed. Once bone was soft, samples were rinsed 5× with ultrapure water, repeatedly sonicated in petroleum ether for five-minute intervals until all visible lipids were removed, rinsed and sonicated twice in ultrapure water, and freeze-dried overnight.

### 2.3. Sample Analysis

All samples were analyzed in the UC Stable Isotope Biogeochemistry Facility on a Costech Elemental Analyzer connected to a Thermo Scientific Delta V IRMS via a Costech Conflo IV interface. For dried, homogenized leaves, we analyzed carbon (both δ^13^C and weight %C) and nitrogen (both δ^15^N and weight %N) separately. We initially weighed ca. 2.0 mg of each sample into tin boats for carbon analysis. We then weighed out between 4 and 10 mg for nitrogen analysis based on the estimated weight %N of each sample obtained during carbon analysis. For small mammal samples, we weighed ca. 0.4 mg of dried collagen or fur keratin into tin boats. Carbon and nitrogen isotope and elemental data were obtained in the same run.

Following Skrzypek [83], we corrected all isotope data for linearity and drift using powdered caffeine, and scale using caffeine and corn starch (for plant carbon), or caffeine and an isotopically spiked glutamic acid (USGS 41) for plant nitrogen and both isotopes for animal tissues. We monitored analytical accuracy using the average difference between measured and known values for two independent reference materials: powdered glycine and soy flour for plants or homogenized gelatin for animal tissues. Accuracy for plant runs was 0.15‰ for carbon and 0.36‰ for nitrogen. Accuracy for small mammal runs was 0.20‰ and 0.19‰ for carbon and nitrogen, respectively. Finally, we calculated precision using the square root of the summed difference between known and measured values for all four reference materials divided by the total number of standards included (N = 52–55 depending on material and isotope analyzed). Estimated precision was 0.19‰ for plant carbon, 0.10‰ for plant nitrogen, 0.10‰ for animal carbon, and 0.09‰ for animal nitrogen. The mean absolute isotopic difference ± one standard deviation (1σ) for samples run in duplicate was 0.21 ± 0.21‰ and 0.47 ± 0.35‰ for plant carbon and nitrogen, respectively (N = 11), and 0.59 ± 0.47‰ and 0.50 ± 0.35‰ for animal tissue carbon and nitrogen, respectively (N = 10).

### 2.4. Data Analysis

We conducted all statistical analyses in JMP Pro 18.0.0 with significance set to α = 0.05. We evaluated homogeneity of variances using Levene tests. Due to small sample sizes and variable data distributions, we compared groups using non-parametric Wilcoxon and Kruskal–Wallis tests, followed by Steel–Dwass All Pair post hoc comparisons when needed.

For plants, we evaluated patterns in δ^13^C, δ^15^N, and atomic C/N. We explicitly included elemental data for plants because the relative concentration of carbon and nitrogen (atomic C/N) in a leaf sample can provide a rough approximation of protein content, and we wanted to be able to evaluate how this might vary among taxa or spatially at BRNI. Raw data for all foliage samples are provided in Table S1. One *Dypsis* sample from T2 had a very low δ^15^N value (−9.2‰), making it a statistical outlier (and does not seem biologically feasible). We, therefore, excluded this datum from all figures, tables, and statistical analyses. As we will present in more detail in the Results, leaves collected along T1 had significantly higher δ^13^C values than those collected along T2 (−31.6 ± 2.5‰ vs. −32.9 ± 2.9‰; T = −2.33, df = 85, *p* = 0.022). Nitrogen isotope values were apparently but insignificantly distinct between transects (−1.4 ± 1.7‰ for T1 vs. −0.8 ± 1.9‰ for T2; T = 1.64, df = 85, *p* = 0.10), and this was also the case for atomic C/N (74.4 ± 31.4 for T1 vs. 66.87 ± 30.0, T = −1.12, df = 83, *p* = 0.27). Based on this information, we decided to evaluate data separately for the two transects. We compared δ^13^C, δ^15^N, and atomic C/N values between transects for the three genera that were sampled in both T1 and T2, as well as among plant genera within each transect, and between forest edge (<900 m from the southern BRNI boundary) and forest interior (>900 m from the southern BRNI boundary), when possible, for individual genera within each transect (*Dypsis* and *Ravenala* from T1 and *Dypsis*, *Pandanus*, and *Ravenala* from T2). We also used linear regressions to investigate potential changes in δ^13^C, δ^15^N and atomic C/N values with increasing distance from the southern edge of BRNI for each transect. We were not able to include all of these variables in a single analysis due to often small and uneven sample sizes.

For small mammals, we only had bone or fur for some individuals and species. We maximized our dataset by combining data from these two tissues. Collagen and keratin reflect different windows of time (months to years versus weeks to months) and have different amino acid compositions, reviewed in [71,72,84]. Consequently, there can be isotopic differences between tissues within the same individual, but as noted above, differences between collagen and keratin should be small (<1‰). Following Crowley et al. [73], we accounted for compositional differences among tissues by applying blanket corrections of −0.9‰ and −0.8‰ to bone collagen δ^13^C and δ^15^N values, respectively, effectively converting them to fur keratin isotope space. These correction factors are based on a broad assortment of wild and captive primates, but we believe they are applicable to our dataset; primates have a generalized physiology and encompass the breadth of diets, life history attributes, and body sizes exhibited by small mammals. We had both fur and bone available for 19 individuals. The average isotopic difference (±1σ) between uncorrected collagen and fur keratin was 1.6 ± 0.8‰ for carbon and 1.0 ± 0.7‰ for nitrogen. These values shifted slightly when using converted collagen values (0.9 ± 0.6‰ and 1.2 ± 0.9‰ for δ^13^C and δ^15^N, respectively). Remaining isotopic differences between keratin and converted collagen may reflect seasonal variability in consumed foods or activity levels [42,85,86,87,88,89,90], although it is also possible that the tissue conversion values we used were not entirely correct. Our combined fur and converted collagen dataset included 60 individuals. For those individuals that had both fur and bone available, we used the averaged δ^13^C and δ^15^N values for fur and converted collagen in all statistical comparisons, figures, and tables. Raw keratin and collagen, as well as converted collagen isotope data for each individual, are provided in Table S2, and summary fur keratin, raw bone collagen, and converted collagen data for each species are provided in Table S3.

Prior to conducting any further statistical analyses, we checked for isotopic differences between collection years. All bats and the single Asian shrew were collected in 2015, but rodents and tenrecs were sampled in both 2015 and 2016. Overall, there was no statistically detectable difference in either δ^13^C or δ^15^N values between years for either order (*p* > 0.05 for all comparisons), and with only a few exceptions, average values differed by <0.5‰ between years for any given species (Table S4). We, therefore, combined data for the two years.

We compared δ^13^C and δ^15^N values among small mammal orders (tenrecs, bats, shrews, rodents), as well as among species within each order, without considering where samples were collected. We ran all analyses, both including and excluding taxa, with a sample size of N = 1. We then investigated potential geographic isotopic patterns at the ordinal level using two different criteria. First, we compared isotope data for animals that were trapped in the forest interior (>900 m from the southern BRNI boundary), forest edge (<400 m from the southern BRNI boundary), or in villages outside of the forest. Second, we examined possible isotopic differences among habitats. At the time when our study was conducted, the vegetation in southern BRNI and the area immediately outside of the reserve’s boundaries varied between undisturbed and slightly degraded moist evergreen forest, second-growth forest (dominated by *Psidium* and *Ravenala*), and anthropogenic (village or agricultural area). When possible, we also evaluated potential differences between sexes for each species in each zone, but we note that, in certain cases, sample sizes were small, so statistical results should be interpreted with caution.

Finally, we plotted summary carbon and nitrogen isotope data (mean ± 1σ) for plants and small mammals in bivariate space to visually assess niche partitioning. For visualization purposes, we converted summary foliage data to “herbivore fur space” by adding 5‰ to plant δ^13^C and 3‰ to plant δ^15^N values, following [67,68]. We acknowledge that tissue–diet offset can vary considerably both within and among taxa depending on diet composition as well as physiology, reviewed in [68,70,71,72,91,92]. Nevertheless, we feel these conversion values are reasonable for visualization purposes as we are only dealing with small-bodied mammals that have relatively similar metabolisms and digestive physiologies and consumed exclusively or predominantly C_3_ foods at BRNI. We included previously published fur data for 28 mouse lemurs (*Microcebus simmonsi*) from BRNI [93]. The lemur samples were collected several years before the other small mammals, but the data still provide a useful visual reference.

## 3. Results

### 3.1. Trends for Vegetation

**Figure 2:**
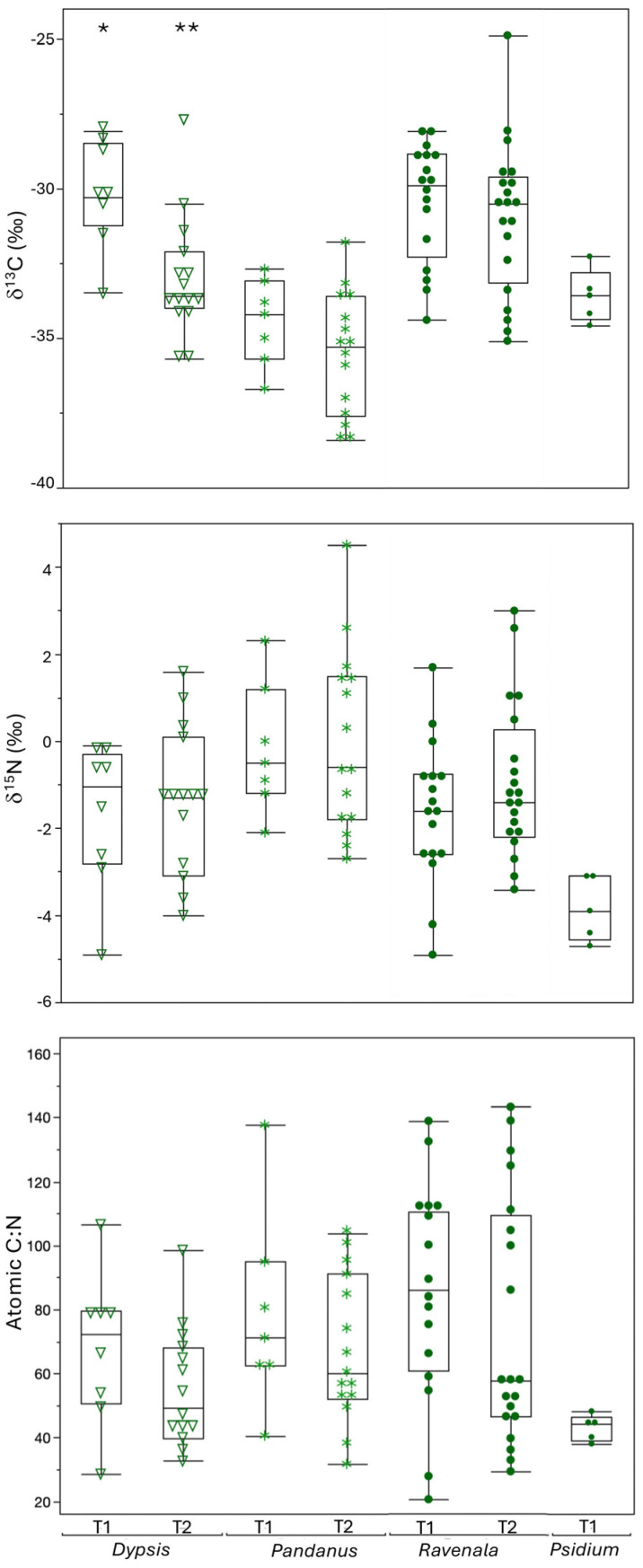
Box plots showing carbon isotope, nitrogen isotope, and atomic C/N data for plant taxa in each transect in the Betampona Réserve Naturelle Intégrale (BRNI). Boxes represent 25 and 75% quartiles, and whiskers contain 1.5-times the interquartile range. Asterisks indicate significant differences between transects for any given genus. The only significant difference is for *Dypsis* δ^13^C values.

Overall, carbon and nitrogen isotopic data for foliage samples ranged from −38.4 to −24.9‰ and −4.9 to +4.5‰, with average and standard deviation values of −32.4 ± 2.8‰ and −1.2 ± 1.9‰, respectively. As noted in the Methods, leaves from T1 had significantly higher median δ^13^C values than those from T2. Looking at specific taxa (Figure 2), *Dypsis* δ^13^C values were significantly higher at T1 than T2 (χ^2^ = 7.28, df = 1, *p* = 0.0070), but there were no statistically significant differences in δ^13^C between transects for either *Pandanus* or *Ravenala*. Nitrogen isotope values and atomic C/N were indistinguishable between transects for all three genera (Figure 2). There were also no differences in variance between transects for either isotope or atomic C/N for any species (Levene *p* > 0.05 for all comparisons).

Comparing plant genera within each transect, isotopic variances were indistinguishable among genera, but there were significant differences in median δ^13^C values among taxa for both T1 and T2 (Figure 2; Table 2). For T1, post hoc analyses indicate *Pandanus* had lower δ^13^C values than *Dypsis* and *Ravenala*, while *Psidium* was statistically indistinguishable from any of the other taxa. For T2, δ^13^C values were statistically indistinguishable for *Dypsis* and *Ravenala*, and *Pandanus* had significantly lower values. There were also differences in median δ^15^N values among plant genera for T1 (Table 2). Post hoc analyses indicate that *Psidium* had significantly lower δ^15^N values than *Pandanus* or *Ravenala* (and visibly but insignificantly lower values than *Dypsis*). There were no differences in median δ^15^N values among taxa at T2, and there were no differences in variance among taxa for either isotope in either transect. Finally, there were significant differences in median atomic C/N among taxa for T1 (Figure 2; Table 2). *Psidium* had visibly smaller values than the other genera, but post hoc tests failed to detect any significant pairwise comparisons. There were no differences in median atomic C/N for T2. Conversely, variance in atomic C/N varied among taxa for T2 but not T1. *Psidium* had only marginally (and insignificantly) less variable atomic C/N than other taxa at T1, while *Ravenala* had significantly more variable C/N than either *Dypsis* or *Pandanus* at T2 (Figure 2; Table 2).

**Figure 3:**
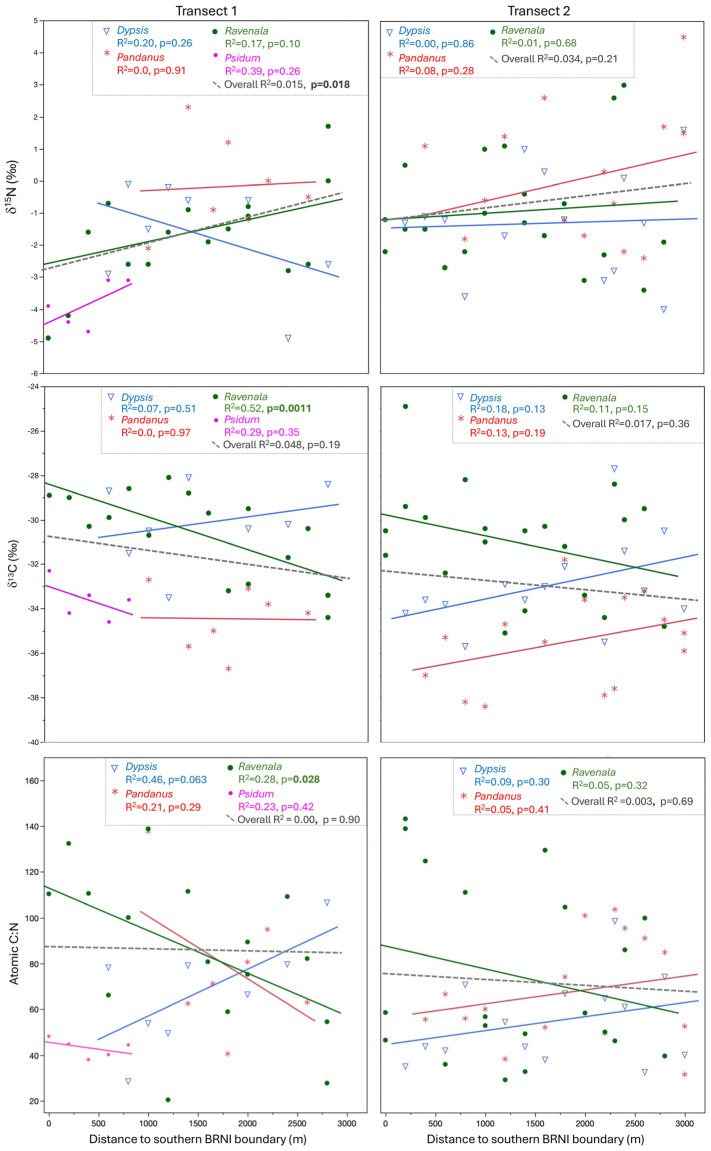
Bivariate plots showing the relationship between distance from the Betampona Réserve Naturelle Intégrale (BRNI) boundary and carbon, nitrogen, and atomic C/N for plants along Transect 1 and Transect 2. Results of regression analyses are provided for all plants as well as individual genera.

Comparing edge (<900 m from the southern BRNI boundary) versus interior (>900 m from the BRNI boundary) samples for individual plant genera in each transect, the only significant difference was for *Dypsis* δ^13^C values along T2 (Table 2). When all plants were included, regressions indicated slight but insignificant decreases in foliar δ^13^C values with increasing distance along both transects (Figure 3). Along T1, there was a significant decrease in δ^13^C values for *Ravenala* and an apparent but insignificant decrease in δ^13^C values for *Psidium*. In contrast, there was no relationship between distance and δ^13^C values for *Pandanus*, and there was a slight, but insignificant, increase in δ^13^C values for *Dypsis*. Along T2, there were no significant trends in δ^13^C values with distance, although there was an apparent (but insignificant) decrease in δ^13^C values for *Ravenala* and an increase for both *Dypsis* and *Pandanus*. Including all plant samples, we observed a significant overall increase in foliar δ^15^N values with increasing distance along T1 but not T2 (Figure 3). There were no significant trends between δ^15^N and distance for individual genera along either transect, and apparent patterns for individual taxa differed both within and between transects. The only significant relationship between atomic C/N and distance for either transect was for *Ravenala* along T1 (atomic C/N declined with distance), but there was also an apparent decline in atomic C/N with distance for *Psidium* along T1 and *Ravenala* along T2. In contrast, there was an apparent increase in C/N with distance for *Dypsis* along both transects, and *Pandanus* showed opposing patterns along T1 and T2 (Figure 3).

### 3.2. Small Mammals

All five tenrecs, two nesomyine rodents (*Eliurus petteri* and *Eliurus webbi*), and the single introduced shrew (*Suncus murinus*) were trapped exclusively in the forest interior (>900 m from the southern BRNI boundary). Two bat species (*Macronycteris commersoni* and *Rousettus madagascariensis*) were only trapped at the forest edge (<400 m from the BRNI boundary), and two bats (*Myotis goudoti* and *Myzopoda aurita*) and two rodents (*Eliurus minor* and *Rattus rattus*) were trapped both in the forest interior and forest edge (Table 2). The fifth bat species, *Mops leucostigma*, was exclusively captured in two villages outside of BRNI (Fontsimavo and Ambodirafia; Figure 1). All tenrecs and rodents as well as the single shrew were either trapped in undisturbed or slightly degraded forest. In contrast, with the exception of *Mops leucostigma*, bats were only trapped in slightly disturbed or second-growth forest. The single *Rousettus* (UADBA SMG-19297) and one *Myzopoda* (FMNH 231934) were captured in second-growth forest just outside of BRNI proper.

**Figure 4:**
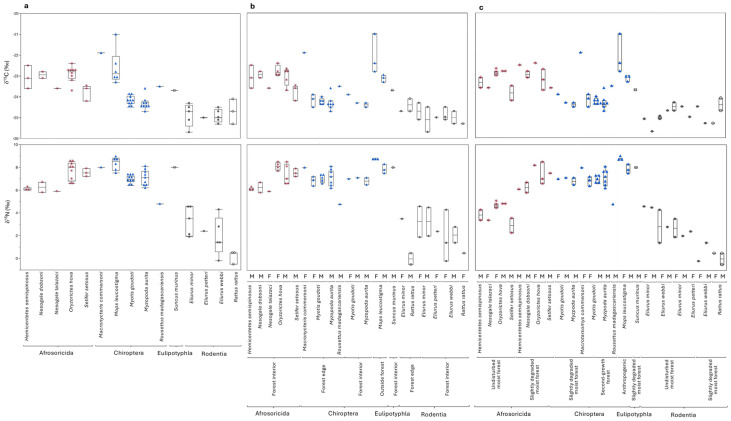
Box plots showing carbon and nitrogen isotope data for: (**a**) each small mammal taxon at Betampona Réserve Naturelle Intégrale; (**b**) males and females of each taxon in forest interior, forest edge, and outside of the forest; and (**c**) males and females of each taxon in undisturbed moist evergreen forest, slightly degraded moist evergreen forest, second-growth forest, or anthropogenic villages and agricultural areas. Boxes represent 25 and 75% quartiles, and whiskers contain 1.5 times the interquartile range. Introduced taxa (*Rattus rattus* and *Suncus murinus*) are represented with hollow symbols.

Including all individuals, there were significant differences in both median δ^13^C and δ^15^N values, as well as isotopic variance among small mammal orders (Figure 4; Table 3). Post hoc tests indicate that tenrecs had higher median δ^13^C values than bats, and both groups had higher δ^13^C values than rodents. Tenrecs and bats had statistically indistinguishable δ^15^N values, and both groups had higher δ^15^N values than rodents. Carbon isotopes were more variable for bats than other groups, while nitrogen isotope values were most variable for rodents. The single shrew was statistically indistinguishable from all other groups; excluding it from the ordinal comparison did not change the significance of the Kruskal–Wallis or Levene tests (or the pairwise differences among the other taxa). Excluding *Mops leucostigma* (which was only trapped well outside of BRNI), the results were very similar, with one exception: variance for δ^13^C was equal among groups (Table 3).

There were apparent but insignificant differences in median δ^13^C values among tenrec species (Figure 4; Table 3). There were significant differences in δ^15^N values, but post hoc tests failed to detect any differences among taxa. Isotopic variance was indistinguishable among species for both isotopes. Excluding the single *Nesogale talazaci* did not affect these results, with one exception: variance for δ^15^N became significantly different (Levene *p* = 0.049; Table 3). *Hemicentetes semispinosus* and *Setifer setosus* had smaller variance than *Nesogale dobsoni* or *Oryzorictes hova*. There were no significant differences in either median isotope values or isotopic variance among rodent species, although the three sampled *Rattus* had apparently lower δ^15^N values than any of the *Eliurus* species (Figure 4; Table 3). Again, excluding the single *Eliurus petteri* did not influence these results, with one exception: median δ^15^N values differed significantly among the other three species, but there were no pairwise differences among taxa (Table 3). In contrast, there were significant differences in both variance and median δ^13^C and δ^15^N values among bat species. Post hoc tests indicate that *Mops leucostigma* had significantly higher and more variable δ^13^C than either *Myotis goudoti* or *Myzopoda aurita*. *Mops leucostigma* also had significantly higher δ^15^N values than *Myotis goudoti*, while *Myzopoda aurita* had the most variable δ^15^N values. The single *Macronycteris commersoni* and *Rousettus madagascariensis* were statistically indistinguishable from other bat species, but this was likely due to small sample size as the two species were visually quite distinct (Figure 4). The *Macronycteris commersoni* isotopically resembled *Mops leucostigma*, while *Rousettus madagascariensis* had uniquely low δ^15^N values (Figure 4; Table 3). Excluding these two taxa with only one sample each did not affect the statistical comparisons for bats (Table 3). However, excluding *Mops leucostigma*, the remaining bat species had statistically indistinguishable median δ^13^C and δ^15^N values, although carbon was nearly significant (Figure 4; Table 3), which was likely driven by the single *Macronycteris*. Differences in variance for carbon isotopes also disappeared but remained for nitrogen isotopes, likely driven by the single *Macronycteris commersoni* and *Rousettus madagascariensis*, which, as noted above, were rather isotopically distinct. There were no differences between *Myotis goudoti* and *Myzopoda aurita* (Figure 4; Table 3).

There were no differences in median δ^13^C values or isotopic variance among rodents captured in the forest edge (<400 m from the southern boundary of BRNI) and the forest interior (>900 m from the boundary; Figure 4; Table 4). Median δ^15^N values were ca. 1.5‰ higher for rodents from the forest interior than the edge, but this difference was not significant. There were also no differences in isotopic variance among zones for bats, but there were significant differences in both median δ^13^C and δ^15^N values (Figure 4; Table 4). There were no isotopic differences between bats trapped in the forest interior or edge, but bats trapped in villages outside of BRNI (exclusively *Mops leucostigma*) had higher δ^13^C and δ^15^N values than bats trapped within BRNI. Four species were captured in both forest edge and interior (*Myotis goudoti*, *Myzopoda aurita*, *Eliurus minor*, and *Rattus rattus*). There were no statistically detectable isotopic differences between distance zones for any of these species (*p* > 0.05 for all comparisons; Figure 4). One *Myzopoda aurita* and two introduced *Rattus rattus* from the forest edge had δ^13^C values that were ca. 1‰ higher than their counterparts in the forest interior, but all other differences were ≤0.3‰ (Figure 4).

There were no differences in median δ^13^C or δ^15^N values for tenrecs trapped in undisturbed and slightly degraded forest (Figure 4; Table 4). There were also no differences in median δ^13^C between these two habitats for rodents. However, rodent δ^15^N values were significantly higher in undisturbed than slightly degraded forest. There were also significant differences in both median δ^13^C and δ^15^N values among habitats for bats (Table 4). These were identical to the patterns described above: bats from slightly degraded forest and second-growth forest were isotopically indistinguishable, while *Mops leucostigma* trapped in villages outside of BRNI had higher values (Figure 4; Table 4). There were no differences in variance among any of these groups.

Five mammal species were trapped in both slightly degraded and undisturbed forest (*Hemicentetes semispinosus*, *Oryzorictes hova*, *Setifer setosus*, *Eliurus minor*, and *Eliurus webbi*), and two species were trapped in both slightly degraded and second-growth forest (*Myotis goudoti* and *Myzopoda aurita*). There were no significant isotopic differences between habitats for any of these species, although there were some small, visually apparent differences for a few taxa (Figure 4). The single *Hemicentetes semispinosus* trapped in slightly degraded forest had a slightly (<1‰) higher δ^13^C value than the two individuals trapped in undisturbed forest. There were larger apparent (but insignificant) isotopic differences between habitats for both *Eliurus minor* and *Eliurus webbi*. Nitrogen isotope values were >1‰ higher for both taxa in undisturbed forest than slightly degraded forest, and δ^13^C was slightly lower in undisturbed forest for *Eliurus minor* (Figure 4).

There were negligible isotopic differences between sexes for most taxa (Figure 4). The single female *Rattus rattus* had a lower δ^13^C value than the two males, but the female was also trapped in the forest interior while both males were trapped in the forest edge. Male *Oryzorictes hova* had more variable δ^15^N values than females in the forest interior, but median values were indistinguishable. In contrast, there were significant differences in both δ^13^C and δ^15^N values between sexes for *Mops leucostigma* trapped in villages (Figure 4). The three females had significantly higher δ^13^C and δ^15^N values than the three males (χ^2^ = 3.86, df = 1, *p* = 0.0495 and χ^2^ = 3.97, df = 1, *p* = 0.046, respectively). Female *Mops leucostigma* also had significantly more variable δ^13^C values than males (Levene *p* = 0.0498). The apparent isotopic differences between undisturbed and slightly degraded moist evergreen forest for *Eliurus minor* and *Eliurus webbi* were maintained at the sex level (Figure 4). We consider possible reasons for small isotopic differences among sexes or habitats for particular species in the Discussion.

**Figure 5:**
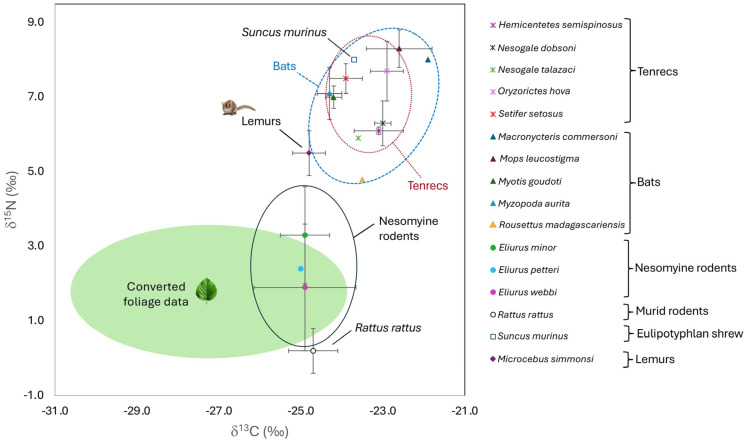
Bivariate plot showing summary (mean ± 1σ) isotope data for understory foliage (converted to animal fur space following [67,68]) and small mammal species from southern Betampona Réserve Naturelle Intégrale (BRNI). Previously published fur isotope data for 28 *Microcebus simmonsi* are from [93]. We note that the converted plant data are approximations and could differ by 1–2‰ from those that are plotted. Nevertheless, these provide a useful visual reference for assessing foraging behavior of small mammals in southern BRNI.

Plotting summary isotope data for each taxon in bivariate isotope space helps illustrate potential niche partitioning among taxa (Figure 5). Overall, there was a considerable amount of isotopic overlap between bats and tenrecs, but small differences and patterns emerge if one examines individual taxa. For example, among bats, the single sampled *Rousettus madagascariensis* and *Macronycteris commersoni* occupied unique isotopic space, and *Mops leucostigma* had higher δ^13^C and δ^15^N values than *Myotis goudoti* and *Myzopoda aurita* (Figure 5). In contrast, *Myotis* and *Myzopoda* had nearly identical isotopic niches. Isotopic niche space for the five tenrec species fell within the middle of the space occupied by bats (Figure 5). *Oryzorictes hova* and *Setifer setosus* had elevated δ^15^N values compared to the other three tenrecs but also occupied distinct δ^13^C space (with higher δ^13^C values for *Oryzorictes* than *Setifer*). *Hemicentetes semispinosus* and *Nesogale dobsoni* had very similar δ^13^C and δ^15^N values, while the single *Nesogale talazaci* had a similar δ^15^N but slightly lower δ^13^C value that only overlapped with *Hemicentetes* (Figure 5). Rodents had lower δ^13^C and δ^15^N values than other small mammal groups. The three endemic *Eliurus* species had nearly identical mean δ^13^C values but distinct δ^15^N values (values were higher for *Eliurus minor* than *Eliurus webbi* or *Eliurus petteri*). There was also relatively large isotopic variability for *Eliurus minor* and *Eliurus webbi*. The single introduced *Suncus murinus* appears to have occupied a distinct isotopic space, with a δ^13^C and δ^15^N value that place it slightly outside of the range for *Setifer setosus*. *Rattus rattus* also occupied a unique isotope space, with considerably lower δ^15^N values and slightly higher δ^13^C values than the three endemic rodents (Figure 5). Finally, previously published data for *Microcebus simmonsi* fall in a unique isotopic space; lemurs had similar δ^13^C values but higher δ^15^N values than rodents and lower δ^13^C values and δ^15^N values than most bats or tenrecs (Figure 5).

## 4. Discussion

We set out to establish an isotopic baseline for southern Betampona Réserve Naturelle Intégrale (BRNI) using foliage from understory vegetation along two transects (T1 and T2) that ran from the forest edge to interior in southern BRNI, as well as indirectly evaluate foraging niches for a variety of small mammals from the same area. The isotopic data reveal both expected and surprising patterns.

### 4.1. Plants

Isotopic data for plants only partially followed the expected trend of decreasing δ^13^C with increasing distance from the southern boundary of BRNI. With the exception of δ^13^C for *Dypsis* along T2, there were no statistically detectable differences between samples collected in the forest edge (<900 m from the southern BRNI boundary) and interior (>900 m from the boundary), and the pattern for *Dypsis* along T2 was the opposite of that expected (values were 1‰ lower closer to the forest edge; Table 2). There was a significant negative relationship between δ^13^C and distance for *Ravenala* along T1 and apparent (but insignificant) negative relationships for *Psidium* along T1, *Ravenala* along T2, and plants overall in both transects (Figure 3). However, *Dypsis* and *Pandanus* did not follow this pattern. Instead, their δ^13^C values either showed no relationship with distance from the BRNI boundary or a slight (but insignificant) positive one. Nitrogen isotope values also showed rather unexpected patterns. With the exception of *Dypsis* along T1, all taxa had apparently (but insignificant) increasing δ^15^N values with increasing distance from the southern BRNI boundary along both transects, and there was an overall significant positive relationship between foliar δ^15^N and distance for T1 (Figure 3). Lastly, atomic C/N, which reflects foliar nitrogen content, also varied considerably among taxa and between transects (Table 2; Figure 3).

Variable (and unexpected) patterns among taxa likely reflect physiological differences as well as where plants were sampled. There were differences in δ^13^C and, to a lesser degree, δ^15^N and atomic C/N values, among plant taxa that were not an artefact of where samples were collected. *Ravenala* consistently had δ^13^C values that were ca. 3‰ higher than *Psidium*, and *Dypsis* consistently had δ^13^C values that were ca. 2–4‰ higher than *Pandanus* sampled at the same distance along transects (Figure 3; Table 2). *Ravenala* also had consistently elevated δ^15^N values compared to co-occurring *Psidium* along T1 (Figure 3), but the more general pattern for nitrogen was increasingly variable values with increasing distance from the BRNI boundary for both transects (Figure 3). These data suggest differences in plant physiology, as well as variable water use efficiencies and access to different forms of nitrogen among taxa, which would not be surprising. *Psidium* is a dicot with a relatively deep root system. The other three genera are all monocots but are phylogenetically not closely related (visual similarities among taxa reflect convergent evolution). Like many true palms in the family Aracaceae, *Dypsis* has a shallow, lateral system of fine roots. *Pandanus*, on the other hand, tends to have exposed prop and stilt roots that help support the trunk, and *Ravenala* has a rhizomatous rooting system similar to other members of the Zingeberales [94,95,96].

Given the physical (and isotopic) differences among taxa, where samples were collected could help account for some of the unexpected spatial patterns we found. In particular, only *Psidium* and *Ravenala* were collected up to the reserve boundary along T1 (Figure 3), and it is conceivable that *Psidium*, which had relatively low carbon and nitrogen values, is responsible for the lack of a significant relationship between δ^13^C and distance from the BRNI boundary when all plants were included from T1 (and the existence of one for δ^15^N). However, *Psidium* cannot be entirely responsible, as the same patterns were observed for *Ravenala*, which was collected along the entirety of T1. It is possible that some of the apparent trends (or lack of them) are due to the relatively small sample sizes, but we think this is also not a satisfactory explanation and another factor must be involved. We explore several possibilities below.

First, it is possible that we included more than one species of *Ravenala*, *Pandanus*, or *Dypsis* in our sampling. Multiple species of these three genera occur at BRNI [78,79,80], and we were not able to identify plants to the species level. Highly variable δ^13^C, δ^15^N, and atomic C/N for *Ravenala* along T2 may support this possibility.

Second, the vegetation in southern BRNI is not pristine. Despite its strict nature preserved status, clear signs of anthropogenic activities are evident within the perimeter zone of the protected area as well as outside its limits. The dominant vegetation along the first 900 m of T1, particularly the presence of *Psidium* and *Aframomum* and dense growths of *Ravenala*, is indicative of a regenerating and at least partially cleared forest habitat. Additionally, within the relatively intact forest zones, numerous canopy gaps are present due to windstorms [34,35].

Third, edges are not new at BRNI, and they may not be particularly abrupt. As documented elsewhere, the age of a forest edge will influence the amount of vegetation regrowth (especially in the understory), which in turn can impact microclimate conditions, as well as the degree to which atmospherically deposited nitrogen gets trapped and incorporated into understory vegetation [60,97]. Both T1 and T2 had variable vegetation cover and included relatively open and disturbed areas. These would likely have resulted in more exposure to sun and wind, as well as lower relative humidity compared to undisturbed forest. We further note that due to the orientation of the trail system and shape of BRNI, a portion of T1 paralleled the western boundary of the reserve, and, consequently, in reality, none of the samples from T1 were collected much more than 1 km from the forest edge (Figure 1). Yet there were also few differences between transects (with the exception of significantly higher δ^13^C values for *Dypsis* from T1 than T2; Figure 2), which suggests that, overall, plants in both transects experienced similar conditions. Woody vegetation is present to varying degrees outside of the BRNI boundary, and this could provide a buffer to understory vegetation near the reserve limit.

Fourth, variable soil composition may also help explain foliar isotope values at BRNI [55]. There are several soils present at BRNI, including haplic acrisols and ferralsols, as well as cambisols [98]. The spatial distribution of these soils is not well defined, but we might expect it to reflect both geology and topography. The geology of the southern portion of BRNI is primarily Paleoarchaen migmatitic orthogneiss, but there are also outcrops of undifferentiated Paleoproterozoic ultramafic igneous rocks [99]. There is considerable topographic variability in southern BRNI, especially along T2 (S.M.G. personal observation). In addition to influencing microclimate conditions, spatially heterogeneous geology, topography and vegetation structure superimposed on a varied history of human disturbance would be expected to influence soil depth and moisture, decomposition rates, and nutrient cycling, and all of these variables could have an influence on both the isotopic composition and elemental composition of plants [56,57,58,59,60,61].

There is still much we do not understand about the relationships between moisture availability, temperature, soil composition and texture, nutrient cycling, and primary productivity, especially in tropical settings [51,52,100,101,102,103,104,105,106,107]. Overall, foliar data at BRNI demonstrate how variable and complex isotopic patterns can be even within a relatively healthy moist tropical evergreen forest setting. Broad generalizations about edge effects on foliar isotopes may be unwise, and more work, especially in tropical settings, would be beneficial.

### 4.2. Small Mammals

Remarkably little is known about the foraging ecology of most of the small mammal taxa we analyzed at BRNI, the majority of which are endemic to Madagascar (Table 1). Isotope data support niche partitioning among orders, as well as among some species within orders (Figure 4 and Figure 5; Table 3 and Table 4). Nitrogen data indicate that, overall, tenrecs, bats, and introduced shrews foraged at a higher trophic level than either endemic nesomyine rodents or introduced *Rattus*, and with the possible exception of several bats (which we will discuss in more detail below), carbon isotope data are consistent with all individuals foraging on C_3_ foods in forested habitat. We acknowledge that sample sizes are small for all of the taxa (and very small for a few species), so statistical tests may not be robust. Nevertheless, we believe the data are informative. For example, relatively low δ^15^N values for all of the sampled rodents would be consistent with primarily herbivorous diets. There were broad isotopic similarities between tenrecs and bats (Figure 4 and Figure 5). However, bats had significantly higher δ^13^C values than tenrecs (Table 3), which likely reflect differences in where the two groups forage. These data would be consistent with the sampled tenrecs primarily foraging on or near the forest floor in the interior of BRNI and bats foraging higher up in the canopy, as well as closer to the forest edge or even outside of BRNI. It seems unlikely that bats and tenrecs routinely consumed the same foods, especially given the expected differences in their preferred prey (Table 1). Nevertheless, it is conceivable that there was some dietary overlap between groups. In particular, *Myzopoda aurita* and *Myotis goudoti* are both small species (<10 g) that are capable of gleaning non-volant arthropods off of vegetation [41,42,43]. We discuss isotopic differences among species within the same order, as well as the possibility of competition between endemic and introduced taxa, below.

#### 4.2.1. Tenrecs

Overall, tenrecs had slightly (ca. 1–2‰) higher δ^13^C values and considerably higher δ^15^N values than converted understory foliage (Figure 5). There were no statistically significant isotopic differences among tenrec species, but sample sizes were quite small for all of the tenrecs except *Oryzorictes hova*. Visually comparing isotopic data, *Hemicentetes semispinosus*, *Nesogale dobsoni*, and *Nesogale talazaci* were relatively tightly clustered (Figure 5), which is rather unexpected given the anticipated differences in their diet and foraging habits. *Hemicentetes semispinosus* is thought to specialize on soft-bodied invertebrates, including earthworms (Table 1). It has very delicate dentition and is probably incapable of consuming hard-bodied invertebrates or vertebrate prey, with the exception of scavenged carrion [108]. In contrast, the two *Nesogale* species have both been observed to predate vertebrates (Table 1). *Nesogale dobsoni* is also unusual in its tendency to eat other tenrecs (especially shrew tenrecs in the *Microgale* genus), and it is unique in its ability to seasonally accumulate and store fat reserves [109]. Yet all three of these species looked very isotopically similar at BRNI, and they had relatively low δ^15^N values (amongst the lowest observed for tenrecs and only slightly higher than mouse lemurs; Figure 5). We hesitate to try to interpret these data more extensively as it is possible the apparent isotopic similarities among taxa are simply an artefact of small sample sizes.

*Oryzorictes hova* and *Setifer setosus* had visually (but insignificantly) higher δ^15^N values than the other three tenrecs and distinct δ^13^C values (Figure 5). Isotopic data for *Oryzorictes hova* partially overlapped with *Mops leucostigma*, while *Setifer setosus* had values that were intermediate to the introduced *Suncus murinus* and the bats *Myzopoda aurita* and *Myotis goudoti* (Table 3; Figure 4 and Figure 5). We suspect that elevated δ^13^C values for *Oryzorictes hova* reflect foraging on insects that consumed grasses outside of forested areas (e.g., grasshoppers), while the lower δ^13^C values for *Setifer setosus* reflect foraging exclusively on forest-dwelling prey, but it is also possible that the elevated δ^13^C values for *Oryzorictes hova* reflect foraging in leaf litter derived from canopy vegetation [47,63,110,111,112]. Similarly elevated δ^15^N values for *Oryzorictes hova* and *Setifer setosus* are also somewhat unexpected as these species are thought to target different kinds of foods. *Setifer setosus* is a trophic omnivore, feeding on both plants and arthropods, including earthworms, while *Oryzorictes hova* is thought to be more faunivorous, primarily consuming insects and earthworms (Table 1). It is possible that elevated δ^15^N values for these two taxa reflect consumption of earthworms, which can have highly variable δ^15^N values depending on what they eat and where they forage in the soil [113,114]. However, because soil δ^15^N is elevated relative to plant δ^15^N, all earthworms would be expected to have relatively elevated δ^15^N values compared to arthropods that forage above ground [112]. Yet, as noted above, *Hemicentetes semispinosus* is also thought to rely heavily on earthworms, and it had relatively low δ^15^N values. Perhaps the three *Setifer setosus* we analyzed consumed vertebrate meat, but this is also not a satisfactory explanation as both *Nesogale* species are also thought to routinely consume vertebrates, and they had relatively low δ^15^N values. All of the sampled tenrecs were captured in undisturbed or slightly degraded moist evergreen forest >900 m from the BRNI boundary (Figure 4), so differences in habitat preferences also cannot explain the data.

We were only able to evaluate differences between sexes for *Oryzorictes hova* (Table 3; Figure 4). A larger sample size would allow us to evaluate possible sex differences more comprehensively and also investigate the degree to which age may influence isotopic data. Most tenrecs are very precocious [85,87], and in general, isotopic differences between nursing mothers and offspring are small (<1‰), reviewed in [115], so age should not have a pronounced effect on our data, but this is not a variable that we were able to control for. Finally, it could be informative to evaluate metabolic influence on tenrec isotope data [116,117]. All tenrecs have relatively low metabolisms, and most undergo seasonal reductions in activity level via daily or seasonal torpor. However, species (and possibly sexes) may vary in the degree to which they rely on torpor, and this can also vary geographically depending on climate conditions [85,87]. Of the species captured at BRNI, *Setifer setosus*, *Hemicentetes* and both *Nesogale* species experience torpor [118,119], but there may be differences in frequency or length of bouts among species, and it is unknown how torpor may differ between males and females.

Previously published data for tenrecs from medium-altitude moist evergreen forest (1440–1550 masl) in the Tsinjoarivo region of central Madagascar provide a useful comparative dataset. Dammhahn and colleagues [120] evaluated potential niche partitioning among multiple tenrec species at two forest parcels. Most of the tenrecs sampled at Tsinjoarivo were *Microgale* species that are not known to occur at BRNI [121], but four tenrecs were included in both studies: *Setifer setosus*, *Nesogale dobsoni*, *Oryzorictes hova*, and *Hemicentetes semispinosus*. At Tsinjoarivo, the single *Setifer setosus* had lower δ^13^C values than other ground-dwelling or fossorial tenrecs and the lowest δ^15^N value for any tenrec in the study (−24.1 and 5.5‰, respectively). Dammhahn and colleagues [120] argued this is because *Setifer setosus* is omnivorous. However, given how different the patterns are between Tsinjoarivo and BRNI, it would seem *Setifer setosus* has some dietary flexibility. Moreover, although this species is typically considered terrestrial, it has been observed to climb (Table 1), and at Tsinjoarivo, its δ^13^C values were comparable to those observed for more scansorial tenrecs.

*Nesogale dobsoni* was much better represented in the dataset from Tsinjoarivo. The seven sampled individuals had average δ^13^C values (−24.5 ± 0.4‰) that were slightly (ca. 0.5‰) lower and δ^15^N values (6.2 ± 0.8‰) that were ca. 1‰ higher than co-occurring *Setifer setosus* [120]. The authors categorized *Nesogale dobsoni* as scansorial and noted that all of the sampled scansorial tenrecs at Tsinjoarivo had comparable δ^15^N values but variable δ^13^C values, which they interpreted to indicate foraging at different heights from the forest floor. They noted that, counterintuitively, the scansorial tenrec with the best adaptations for climbing (*Microgale majori*) had the lowest δ^13^C values but decided that this could be explained if the other sampled scansorial species, including *Nesogale dobsoni*, foraged on prey in leaf litter on the forest floor that was derived from the canopy. At BRNI, *Nesogale dobsoni* also had relatively low δ^15^N values but slightly higher δ^13^C values than some of the other tenrecs, including *Nesogale talazaci*. Perhaps these values also reflect foraging on arthropods in leaf litter, and differences in where taxa foraged above the forest floor could explain slight differences in δ^13^C values between the two *Nesogale* species at BRNI, but we also do not feel secure in making these kinds of data interpretations without a larger sample size.

*Hemicentetes semispinosus* and *Oryzorictes hova* at Tsinjoarivo had higher δ^13^C and δ^15^N values than both *Setifer setosus* and *Nesogale dobsoni*. Similar to BRNI, average δ^13^C for eight *Hemicentetes semispinosus* (−22.9 ± 0.8‰; N = 8) differed by <0.1‰ from the single *Oryzorictes hova* at Tsinjoarivo. However, in contrast to BRNI, δ^15^N values for these two taxa were also nearly identical (ca. 7.3‰). Both of these species are typically considered to be fossorial (Table 1), although Dammhahn et al. [120] categorized *Hemicentetes semispinosus* as ground dwelling, which may be more appropriate. At BRNI, *Hemicentetes* certainly appears to have consumed foods that were isotopically distinct from *Oryzorictes hova*, and it plots more closely to the two *Nesogale* species, especially *Nesogale dobsoni* (Figure 5), which are also considered to be primarily terrestrial, but again, larger sample sizes are needed to evaluate this comprehensively.

Thus, overall, the isotope data provide support for niche partitioning among co-occurring tenrecs but also suggest some surprises given the kinds of foraging behavior we had anticipated. While we expect there is some dietary plasticity among taxa (and sites), there may be other factors at play that we have not been able to evaluate due to limited sample sizes and the complexity of the data. A larger sample size will be needed to draw more concrete inferences about the foraging behavior of tenrecs at BRNI.

#### 4.2.2. Bats

The combination of types of food consumed and where bats forage likely explains isotopic differences among the bat species [110,122]. Relatively low δ^15^N for the single *Rousettus madagascariensis* compared to other sampled bats could readily be explained by a predominantly frugivorous diet. Yet the individual we sampled had considerably higher δ^13^C and δ^15^N values than converted understory foliage or any of the sampled rodents (Figure 5). We expect that this bat regularly visited trees in agricultural areas outside the BRNI boundary. This species routinely forages outside of protected areas elsewhere in Madagascar [123,124].

Elevated δ^13^C and δ^15^N for *Mops leucostigma* and *Macronycteris commersoni* could either support these species foraging on arthropods in the canopy or in agricultural areas beyond the boundaries of BRNI. We cannot disentangle these possibilities with our present dataset but note that all of the sampled *Mops leucostigma* were collected outside of BRNI, and the single *Macronycteris* was sampled at the southern edge of BRNI (Table S2). Both species are widespread, and previous work suggests that all of the bat species we sampled prefer to forage in forest edges, as well as outside of forests [31,42,123,125], perhaps because invertebrate prey is more abundant than in forest interiors [126]. Additionally, both *Mops leucostigma* and *Macronycteris commersoni* predominantly consume beetles (Coleoptera), which can themselves have highly variable isotopic signatures [122], reflecting their variable diets (ranging from herbivore to faunivore to scavenger) and capacity to fly relatively far distances, including outside of forested areas, reviewed in [127,128].

In contrast, *Myzopoda aurita* and *Myotis goudoti* had similar δ^13^C and δ^15^N values that were lower than the other bats (and, as noted above, similar to some tenrecs; Figure 5). Isotopic similarities between these two species are rather remarkable given expected differences in their diets. There were no isotopic differences between individuals that were trapped in the forest edge versus interior or among habitat zones for either species, which is probably a reflection of foraging across a range of habitats, possibly including outside of forest. Perhaps this is a seasonal signal, and there is some dietary overlap between the two species in response to the availability of preferred invertebrate prey during certain months. In western Madagascar, Rakotoarivelo and colleagues [42] found that *Myotis goudoti* diet was dominated by Coleoptera during warmer months, while Lepidoptera and Aranae (spiders) contributed more during the cooler months. In contrast, *Myzopoda aurita*, which is restricted to eastern moist evergreen forests, appears to primarily consume lepidopterans, regardless of the season [43,125]. Lepidopterans should have relatively low δ^13^C and δ^15^N values compared to other arthropods [122] and we, therefore, speculate that the isotope data may be reflecting a similar lepidopteran-based diet for both *Myotis goudoti* and *Myzopoda aurita.* A larger sample size that clearly reflects different seasons would allow us to further test this suggestion and would also enable evaluation of possible seasonal variability in foraging behavior for the other sampled bats [86,88,90].

We were only able to evaluate possible sex differences for *Myotis goudoti* and *Mops leucostigma.* Male and female *Myotis goudoti* did not differ isotopically, regardless of where they were trapped at BRNI (Figure 4), suggesting they ate similar foods. Dietary differences for male and female *Myotis goudoti* have not been previously evaluated, but investigations of other *Myotis* species outside of Madagascar provide mixed support for dietary differences between sexes [129,130,131]. In contrast, *Mops leucostigma* females had higher δ^13^C and δ^15^N values than males at BRNI. This is almost certainly related to differences in preferred prey (this species shows sexual dimorphism in body size; Table 1) but may also reflect differences in where males and females forage. Fecal analysis for this species at Ivoloina, which is ca. 20 km to the southeast of BRNI, suggests that, at least during the rainy season, males and females target different prey [132]. Coleoptera was the most abundant prey in feces collected from males, while Hymenoptera (bees, wasps, and ants) was the most abundant prey for females. We might expect dietary differences among sexes for the other bat species based on body size, or segregation of where males and females forage and roost [90,125], but cannot evaluate this with our current dataset for BRNI.

In summary, isotopic data for bats are, overall, compatible with species targeting different prey and may also support species (and possibly sexes in the case of *Mops leucostigma*) foraging on different foods or in different places within and outside of BRNI, but sample sizes are too small to be able to dive deeper into interpretations with much confidence. Published isotope data for bats elsewhere in Madagascar [122,133,134] can help provide some context for interpreting the limited data from BRNI. In particular, Dammhahn and Goodman [134] analyzed four of the same taxa we analyzed (all but *Myzopoda aurita*) from dry deciduous forest at Ankarana in northern Madagascar. Similar to what we observed at BRNI, *Rousettus madagascariensis* had relatively low median δ^13^C and δ^15^N values (ca. −22.2‰ and 6.9‰, respectively), which the authors interpreted to indicate frugivory in the mid-upper canopy, while *Mops leucostigma* had elevated median δ^13^C and δ^15^N values (ca. −18.6‰ and 10.1‰, respectively) and considerably more variable δ^13^C values, which the authors interpreted as a preference for foraging in open areas [134]. However, in contrast to the patterns we observed at BRNI, *Macronycteris commersoni* had relatively low median δ^13^C and δ^15^N values (ca. −21.7‰ and 8.2‰, respectively), which were only slightly higher than *Rousettus madagascariensis* and lower than any of the other insectivorous taxa, and *Myotis goudoti* had relatively low δ^13^C and elevated δ^15^N values compared to the other insectivorous taxa (−22.2‰ and 10.8‰, respectively). As noted above, both *Macronycteris commersoni* and *Myotis goudoti* have highly variable diets, so it is perhaps not surprising to find spatially variable isotopic patterns for some bats. Dammhahn and Goodman [134] considered both *Macronycteris commersoni* and *Myotis goudoti* to be “low canopy” insectivores, but the isotope data clearly indicate these two species consumed different things, both at Ankarana and BRNI. As discussed above, low δ^13^C values for *Myotis goudoti* would be consistent with this species feeding in the understory at BRNI, while the elevated δ^13^C for the single *Macronycteris* is more suggestive of foraging outside the forest.

#### 4.2.3. Endemic Rodents

Carbon isotope data for the three *Eliurus* species were nearly identical (Table 3) and also similar to mouse lemurs (*Microcebus simmonsi*; Figure 5). All four taxa plotted at the higher end of converted foliage δ^13^C values (Figure 5). We believe these data are compatible with all four species foraging at a similar height from the forest floor in the understory, especially if they primarily consumed fruit, as fruit tends to have slightly (ca. 1‰) higher δ^13^C values than foliage [63,64,66]. As noted above, all three *Eliurus* species had relatively low δ^15^N values compared to the other small mammals, including mouse lemurs, but *Eliurus minor* had higher δ^15^N values than either *Eliurus petteri* or *Eliurus webbi* (and higher than converted foliage values). This suggests the sampled *Eliurus minor*, at least on occasion, consumed some animal matter at BRNI (Table 1). Different foraging strategies are also supported by notable morphological differences in foot structure, as well as cranio-dental measurements and body masses [135]. There were no isotopic differences between individuals trapped in forest edge and forest interior (Figure 4), but both *Eliurus minor* and *Eliurus webbi* had higher δ^15^N values in undisturbed than slightly disturbed moist forest (unlike the more faunivorous bats or tenrecs). This may reflect foraging on more animal matter in undisturbed forest, but that would be contradictory to research in isolated moist forest patches elsewhere that has found primarily frugivorous mammals may increase their consumption of arthropods in more disturbed habitats and near forest edges [63,136]. Baseline shifts in δ^15^N for vegetation among habitat types, or consumption of fruits and seeds from different plant species, could be alternative explanations. We note that while there were negligible isotopic differences between sample years for most taxa, average δ^15^N values were >2‰ higher in 2015 than 2016 for both *Eliurus minor* and *Eliurus webbi* (Table S4). This may reflect annual or seasonal differences in diet for these taxa, but it is hard to disentangle potential temporal patterns from other variables such as space or sex.

Researchers have previously evaluated niche partitioning among co-occurring *Eliurus* species in forest fragments in the Tsinjoarivo region [120], as well as a separate isolated forest fragment at Ambohitantely in central highland Madagascar [75], and these studies can provide some useful comparative data for corroborating our interpretations for *Eliurus* behavior at BRNI. At Tsinjoarivo, three species were sampled (*Eliurus majori*, *Eliurus minor*, and *Eliurus grandidieri*) [120]. There were differences in both δ^13^C and δ^15^N values among these species (*Eliurus minor* had relatively elevated δ^13^C values and lower δ^15^N values, *Eliurus majori* had lower δ^13^C values and intermediate δ^15^N, and the single *Eliurus grandidieri* had intermediate δ^13^C and relatively elevated δ^15^N values). All three species had δ^13^C values that were similar to, or lower than, those for co-occurring tenrecs, and lower δ^15^N values than tenrecs, although data for *Eliurus grandidieri* were less than 1‰ lower than *Setifer setosus* (which the authors suggested may indicate consumption of animal matter).

Published isotopic data exist for plants in the Tsinjoarivo region, including one of the fragments where some of the *Eliurus* were collected [63]. Accounting for expected isotopic differences between herbivores and diet [67,68], converted plant δ^13^C values are compatible with all three of the sampled *Eliurus* species foraging in the forest understory at Tsinjoarivo, which is similar to what we observed at BRNI. However, in contrast to BRNI, converted average δ^15^N value for plants was 1.5–3.5‰ lower than any of the rodents at Tsinjoarivo. It is possible a larger offset would be more appropriate, but we think it is more likely that the sampled individuals (especially *Eliurus majori* and *Eliurus grandidieri*) consumed some animal matter at Tsinjoarivo.

Two species were sampled at Ambohitantely: *Eliurus minor* and *Eliurus majori* [75]. There were no statistically detectable differences in either δ^13^C or δ^15^N between species, although *Eliurus minor* had slightly less variable and, therefore, slightly (ca. 1‰) higher average δ^13^C values (ca. −23 vs. −22‰). There were also no differences in δ^13^C or δ^15^N values between males and females for either species or between *Eliurus majori* trapped in the forest interior and a single individual from the forest edge.

Collectively, these data suggest that *Eliurus* species likely forage primarily in the lower to middle understory, and that they are primarily vegetarian, but there may be dietary differences among co-occurring species. Given how little we know about diet for any *Eliurus* species, a broader evaluation of niche partitioning among co-occurring species, as well as possible dietary flexibility among localities and across seasons, would be very informative.

#### 4.2.4. Introduced Taxa

It has previously been proposed that *Suncus murinus* may compete with small tenrecs (principally *Microgale*), and *Rattus rattus* may compete with larger, semi-arboreal nesomyine rodents (like *Eliurus webbi*) for food [137,138,139]. Introduced *Suncus murinus* and *Rattus rattus* occupied relatively unique isotopic space at BRNI, but our sample sizes were small, especially for *Suncus murinus* (N = 1). Isotope data for *Suncus murinus* more closely resembled bats and tenrecs than rodents, which is what we would expect for an insectivorous or faunivorous animal. Yet the data also suggest *Suncus murinus* foraged on foods that were isotopically distinct from the sampled tenrecs (Figure 5). *Suncus* most closely resembled *Setifer setosus*, but these two would be expected to target different foods based on size and external morphology [21]. In contrast, *Rattus* had comparable δ^13^C values to endemic rodents and the lowest δ^15^N values in our dataset. Indeed, at BRNI, *Rattus rattus* was most isotopically similar to *Eliurus webbi*. However, only one female *Eliurus webbi* (FMNH 232545) had a comparably low δ^15^N value (Figure 4; Table S2). Moreover, *Rattus rattus* at BRNI had lower δ^15^N values than converted understory foliage, indicating a primarily, if not exclusively, herbivorous diet, which is unexpected as this species is typically considered to be a generalist omnivore. At Ambohitantely, *Rattus rattus* rats had comparable δ^13^C values, and higher δ^15^N values (elevated by 2–8‰), than co-occurring *Eliurus minor* and *Eliurus majori*, which is more consistent with expectations for an omnivore [75]. Reported fur δ^13^C and δ^15^N values for *Rattus rattus* from natural forest elsewhere in eastern Madagascar are highly variable (ranging from ca. −25 to > −15‰ and 0‰ to >10‰, respectively). However, out of a dataset of over 500 individuals, only 4 had δ^13^C and δ^15^N values that were comparably low to those we sampled at BRNI, and all of them came from degraded natural forest habitat surrounded by agricultural areas near a village called Ambalafary [12,140]. What is responsible for such low δ^15^N values remains elusive and would be worth exploring in a future study. Overall, the isotopic data suggest introduced small mammals may not be competing with endemic small mammals for food at BRNI, but we caution that both *Rattus rattus* and *Suncus murinus* have highly flexible diets, and our sample sizes were small. The few individuals we analyzed may not be representative of either species in or around BRNI. Further sampling is required.

## 5. Conclusions

In conclusion, our isotopic investigation of plants and small mammals at Betampona Réserve Naturelle Intégrale (BRNI) in eastern Madagascar has yielded interesting results. These results should be viewed as preliminary. We caution that our sample sizes were very small for some taxa, and more work is needed to be able to fully flesh out dietary variability and possible resource competition among endemic taxa, as well as between endemic and introduced taxa. Nevertheless, isotopic data suggest niche partitioning among small mammals at BRNI and fill a previous gap in the growing body of isotopic data available for Madagascar [12]. We did not find a clear isotopic influence of proximity to the forest edge on vegetation, and there were negligible isotopic differences among distance or habitat zones for small mammals at BRNI. The data also suggest that *Suncus murinus* and especially *Rattus rattus* may occupy unique isotopic niche space despite morphological and life history traits that are similar to tenrecs and nesomyine rodents, respectively [137,138,139]. There is ample opportunity for further investigation of foraging niches and resource partitioning among small mammals at BRNI, including the possible influences of geography, season, sex, age, and activity level/metabolism. We stress that animals need not be euthanized for this kind of research. Fur and feces can be sampled with minimal adverse effects on an individual. However, it will be important to account for geographic baseline isotopic variability (e.g., using plants). Without these comparative data, it will not be possible to directly compare isotope data for consumers from different localities.

## Figures and Tables

**Table 1:** Summary of phylogenetic and ecological details for sampled small mammals from Betampona Réserve Naturelle Intégrale and synthesized from [16,17,18,19,20,21,22,23,24,25,26,27,28,29,30,31]. References to altitude are meters above sea level (masl). E = endemic to Madagascar.

Order	Family/ Subfamily	Genus and Species	Mass (in Grams) Average (Range) No. of Individuals	Details	Habit	Habitat	Other Notes
Afrosoricida	Tenrecinae	*Hemicentetes semispinosus* (E)	133.9 (103–184) N = 14	Entomophage *** Soft invertebrates in leaf litter, primarily annelid worms.	Terrestrial (fossorial)	Primary and degraded moist forest, agricultural areas, and villages in eastern Madagascar from sea level up to 1550 masl.	Excavate burrows up to 15 cm below ground level. Social, sometimes living in family groups > 20 individuals.
	Tenrecinae	*Setifer setosus* (E)	247.0 (230–280) N = 5	Omnivore Annelid worms, Orthoptera, ants, fruits, carrion, mollusks.	Primarily terrestrial but also scansorial	Widespread in all forest types, including degraded forest and open habitats from sea level up to 2250 masl.	Tends to be solitary. Mostly forages on ground; sleeps underground in short tunnels it digs.
	Oryzorictinae	*Nesogale dobsoni* * (E)	25.5 (30.5–30.0) N = 7	Carnivore Invertebrates (Orthoptera and occasionally annelids), frogs, and sometimes smaller *Microgale* species.	Terrestrial to scansorial	Intact and disturbed moist evergreen forest, as well as agricultural areas and other non-forested areas from sea level up to 2500 masl.	Captive observations similar to *Nesogale talazaci*. Mostly thought to forage in leaf litter but scansorial behavior supported by a few individuals that were trapped 1–2.5 m above the ground. Thought to be solitary but nest in male/female pairs in captivity. Unique among tenrecs in ability to seasonally store fat reserves in the tail.
	Oryzorictinae	*Nesogale talazaci* * (E)	37.4 (25.0–45.5) N = 18	Carnivore Small vertebrates like frogs as well as arthropods.	Primarily terrestrial	Low altitude to montane moist evergreen intact and disturbed forest (from ca. 800 to 2300 masl).	Primarily forages in leaf litter, under fallen branches, and among roots in captivity. Thought to be solitary but nest in male/female pairs in captivity. Can be spatially clumped within forests.
	Oryzorictinae	*Oryzorictes hova* (E)	42.5 (30.0–15.5) N = 35	Entomophage *** Grasshoppers and soil invertebrates, especially annelid worms.	Terrestrial (fossorial)	Moist and sclerophyllous forest, swamps, forest, secondary vegetation and rice paddies near forests from sea level to 1990 masl.	Virtually nothing known about diet. What we do know is based on stomach contents and feeding in captivity.
Chiroptera	Hipposideridae	*Macronycteris commersoni* ** (E)	Males 73.2 (51.5–98.0) N = 13 Females: 45.1 (39.5–50.0) N = 5	Entomophage *** Large variety of insects but predominantly Coleoptera. Suspected to eat small frogs in southeastern Madagascar, but this has not been directly verified.	Volant	Widespread in both intact and degraded forest across Madagascar. Found in the ecotone between forest and agricultural areas.	This is the largest insectivorous species on Madagascar.
	Vespertillonidae	*Myotis goudoti* (E)	6.0 (4.2–9.2) N = 37	Entomophage *** Varies among studies; Coleoptera, Isoptera, Lepidoptera, Hymenoptera, and Araneae all noted, and in western Madagascar, relative importance of different groups may shift seasonally.	Volant	Widespread in a variety of habitats from sea level to 1600 masl, including intact and degraded low elevation and montane forest, open grassy areas, and agricultural regions.	May roost with other bats (particularly *Minopterus*). Tends to be active earlier in the evening than other bats.
	Pteropodidae	*Rousettus madagascariensis* (E)	Males: 61.1 (49.0–87.0) N = 36 Females: 52.0 (30.5–77.0) N = 32	Herbivore (frugivore) Known to forage on the fruits of native and introduced trees; an important seed disperser.	Volant	Broadly associated with forests and caves. Widespread in multiple forest types as well as agricultural areas, but absent from vast treeless areas.	Likely an important pollinator for *Ravenala*.
	Myzopodidae	*Myzopoda aurita* (E)	8.3 (6.7–10.5) N = 22	Entomophage *** Primarily Lepidoptera as well as some Coleoptera and cockroaches.	Volant	Widespread in a variety of habitats ranging from intact to degraded and fragmented forest, marsh, agricultural areas, and rice paddies at low to mid elevations in eastern Madagascar.	Has unique “horseshoe-shaped” adhesive disks on hands and feet. Appears closely tied to places where *Ravenala* grows.
	Molossidae	*Mops leucostigma*	Males: 22.5 (17.0–28.0) N = 275 Females: 20.1 (16.0–24.0) N = 102	Entomophage *** Coleoptera, Hemiptera, Lepidoptera, and Diptera.	Volant	Occurs on Madagascar and neighboring islands. Widespread in intact and degraded forest, ecotones, agricultural, and even urban areas that are near forest with the exceptions of the extreme south, and elevations > 1200 m in central Madagascar.	
Eulipotyphla	Soricidae	*Suncus murinus*	29.3 (17.0–45.5) N = 19	Omnivore Small mammals, arthropods, plant material.	Terrestrial and fossorial	Introduced and widespread in nearly every environment, including anthropogenic settings.	
Rodentia	Nesomyidae	*Eliurus minor* (E)	35.1 (21.5–49.5) N = 25	Frugivore and granivore Diet presumed based on behavior for other *Eliurus* taxa.	Primarily arboreal, but also terrestrial	Moist evergreen forest from sea level up to 1875 masl. Potentially tolerant of some disturbance.	
		*Eliurus petteri* (E)	74.0 N = 1	Frugivore, insectivore, and granivore Diet presumed. Weak incisors suggest less reliance on hard seeds than other *Eliurus* species.	Primarily terrestrial and perhaps partially arboreal	Restricted to moist forest in central Eastern Madagascar from ca. 400 to 1000 masl.	Not thought to live in sympatry with other *Eliurus* species, but other species present in localities near those where *E. petteri* found.
		*Eliurus webbi* (E)	70.1 (54.0–90.0) N = 19	Granivore (and some frugivory and insectivory) Gnaws holes in shells to extract endocarp. Frugivory and insectivory inferred based on behavior for other *Eliurus* taxa.	Primarily arboreal, but also terrestrial	Intact and degraded low elevation to montane evergreen moist forest in eastern Madagascar, mostly from sea level to 800 masl, but occasionally up to ca. 1500 masl.	Observed to store seeds in its burrows. Granivory inferred based on gnaw holes in seeds stored in burrows. Frugivory and insectivory presumed, based on behavior for other *Eliurus* species.
	Muridae	*Rattus rattus*	105.7 (86.0–134) N = 10 ****	Omnivore and granivore Eats a wide variety of foods.	Scansorial	Widespread globally. Introduced to Madagascar and broadly distributed, from sea level up to ca. 2500 masl in both anthropogenic and natural settings.	

* Until recently, both *Nesogale dobsoni* and *Nesogale talazaci* were included in the larger genus *Microgale*. On the basis of molecular data, they were recently transferred to *Nesogale* [32]. ** *Macronycteris commersoni* was previously placed in the genus *Hipposideros* but this was changed based on molecular data [33]. *** We have used “entomophage” rather than “insectivore” to describe diets of most taxa because this is a more comprehensive term (and includes things like arachnids and worms). **** We have reported body mass values for rats from the Andringitra protected area in central Madagascar [19] but note that larger masses have been reported for rats elsewhere.

**Table 2:** Summary isotope and elemental data for plant genera from each transect at Betampona Réserve Naturelle Intégrale (BRNI), as well as results of non-parametric statistical comparisons. Significant results are presented in bold font. Genera that share a superscript letter within each transect were statistically indistinguishable based on Steel–Dwass All Pairs post hoc analyses. When relevant, data are also presented for samples collected within the forest edge (<900 m from the southern BRI boundary) or forest interior (>900 m from the southern BRI boundary). The only statistically detectable difference was for *Dypsis* δ^13^C values at T2.

Transect	Taxon				δ^13^C (‰)					δ^15^N (‰)					Atomic C:N		
			N	Mean ± 1σ	Median	Min	Max	N	Mean ± 1σ	Median	Min	Max	N	Mean ± 1σ	Median	Min	Max
Transect 1	*Dypsis*	Total	8	−30.2 ± 1.8	−30.3 ^ab^	−33.5	−28.1	8	−1.7 ± 1.7	−1.1 ^ab^	−4.9	−0.1	8	67.8 ± 23.7	72.4 ^a^	28.6	106.6
		Edge (<900 m)	2	−30.1 ± 2.0	−30.1	−31.5	−28.7	2	−1.5 ± 2.0	−1.5	−2.9	−0.1		53.5 ± 35.1	53.5	28.6	78.3
		Interior (>900 m)	6	−30.2 ± 1.9	−30.3	−33.5	−28.1	6	−1.7 ± 1.8	−1.1	−4.9	−0.2		72.6 ± 20.8	72.9	49.6	106.6
			Edge vs. Interior comparison		χ^2^ = 0.11, df = 1, *p* = 0.74					χ^2^ = 0.11, df = 1, *p* = 0.74					χ^2^ = 1.0, df = 1, *p* = 0.32		
	*Pandanus*	Total	7	−34.5 ± 1.4	−34.2 ^c^	−36.7	−32.7	7	−0.2 ± 1.5	−0.5 ^a^	−2.1	2.3	7	78.7 ± 31.0	71.2 ^a^	40.6	137.7
		Interior (>900 m)	7	−34.5 ± 1.4	−34.2	−36.7	−32.7	7	−0.2 ± 1.5	−0.5	−2.1	2.3	7	78.7 ± 31.0	71.2	40.6	137.7
	*Ravenala*	Total	16	−30.2 ± 1.8	−30.3 ^b^	−33.5	−28.1	16	−1.8 ± 1.6	−1.6 ^a^	−4.9	1.7	16	85.6 ± 34.1	85.8 ^a^	20.5	138.8
		Edge (<900 m)	5	−29.3 ± 0.7	−29.0	−30.3	−32.3	5	−2.8 ± 1.8	−2.6	−4.9	−0.7	5	103.9 ± 24.2	110.4	66.2	132.4
		Interior (>900 m)	11	−31.2 ± 2.1	−30.7	−34.4	−28.1	11	−1.3 ± 1.3	−1.5	−2.8	1.7	11	77.2 ± 35.6	80.8	20.5	138.8
			Edge vs. Interior comparison		χ^2^ = 2.70, df = 1, *p* = 0.10					χ^2^ = 1.86, df = 3, *p* = 0.17					χ^2^ = 2.0, df = 1, *p* = 0.16		
	*Psidium*	Total	5	−33.6 ± 0.9	−33.6 ^abc^	−34.6	−32.3	5	−3.8 ± 0.7	−3.9 ^b^	−4.7	−3.1	5	43.2 ± 4.0	44.5 ^a^	38.1	48.2
		Edge (<900 m)	5	−33.6 ± 0.9	−33.6	−34.6	−32.3	5	−3.8 ± 0.7	−3.9	−4.7	−3.1	5	43.2 ± 4.0	44.5	38.1	48.2
			T1 Genus comparison		χ^2^ = 17.50, df = 3, ***p* = 0.0005**					χ^2^ = 12.55, df = 3, ***p* = 0.0057**					χ^2^ = 9.18, df = 3, ***p* = 0.027**		
					Levene *p* = 0.27					Levene *p* = 0.53					Levene *p* = 0.068		
Transect 2	*Dypsis*	Total	15	−33.0 ± 2.0	−33.6 ^a^	−35.7	−27.7	14 *	−1.3 ± 1.7	−1.25	−4.0	1.6	14 *	54.8 ± 18.8	49.2 ^a^	32.7	98.6
		Edge (<900 m)	4	−34.3 ± 1.0	−34.0	−35.7	−33.6	4	−1.8 ± 1.3	−1.5	−2.8	1.7	4	48 ± 15.7	43.0	35.2	70.8
		Interior (>900 m)	11	−32.5 ± 2.1	−33.0	−35.5	−27.7	10 *	−1.1 ± 1.9	−1.3	−4.0	1.6	10 *	57.5 ± 20.0	57.9	32.7	98.6
			Edge vs. Interior comparison		χ^2^ = 4.1, df = 1, ***p* = 0.043**					χ^2^ = 0.18, df = 1, *p* = 0.67					χ^2^ = 0.32, df = 1, *p* = 0.57		
	*Pandanus*	Total	15	−35.5 ± 2.0	−35.3 ^b^	−38.4	−31.8	15	−0.01 ± 2.1	−0.6	−2.7	4.5	15	67.6 ± 22.9	60.2 ^a^	31.7	103.7
		Edge (<900 m)	3	−36.8 ± 1.5	−37.0	−38.2	−35.3	3	−1.1 ± 2.0	−1.8	−2.7	1.1	3	59.5 ± 6.2	56.1	55.7	66.7
		Interior (>900 m)	12	−35.1 ± 2.0	−34.9	−38.4	−31.8	12	0.3 ± 2.1	−0.2	−2.4	4.5	12	69.6 ± 25.3	67.2	31.7	103.7
			Edge vs. Interior comparison		χ^2^ = 1.69, df = 1, *p* = 0.19					χ^2^ = 1.69, df = 1, *p* = 0.19					χ^2^ = 0.083, df = 1, *p* = 0.77		
	*Ravenala*	Total	20	−31.0 ± 2.5	−30.5 ^a^	−35.1	−24.9	20	−0.9 ± 1.8	−1.4	−3.4	3.0	20	74.8 ± 38.4	57.7 ^a^	29.3	143.2
		Edge (<900 m)	7	−29.6 ± 2.5	−29.9	−32.4	−24.9	7	−1.5 ± 1.0	−1.5	−2.7	0.5	7	94.2 ± 45.7	111.1	36.1	143.2
		Interior (>900 m)	13	−31.8 ± 2.3	−31.0	−35.1	−28.4	13	−0.6 ± 2.0	−1.0	−3.4	3.0	13	64.3 ± 30.8	53.0	29.3	129.5
			Edge vs. Interior comparison		χ^2^ = 2.77, df = 1, *p* = 0.096					χ^2^ = 0.98, df = 1, *p* = 0.32					χ^2^ = 2.39, df = 1, *p* = 0.12		
			T2 Genus Comparison		χ^2^ = 20.75, df = 2, ***p* < 0.0001**					χ^2^ = 2.8, df = 2, *p* = 0.25					χ^2^ = 2.26, df = 2, *p* = 0.32		
					Levene *p* = 0.64					Levene *p* = 0.39					Levene ***p* = 0.0003**		

* One *Dypsis* from T2 with a very low δ^15^N value has been excluded.

**Table 3:** Summary data for small mammals trapped within or near Betampona Réserve Naturelle Intégrale. Bold and underlined *p* values respectively indicate significant and marginally significant results. Statistical differences among orders are indicated with superscript numbers, while differences among species within each order are indicated using superscript letters. Orders that share a superscript number, and species within orders that share a superscript letter are statistically indistinguishable.

Order	Species			δ^13^C (‰)					δ^15^N (‰)			
		N	Mean	± 1σ	Median	Min	Max	Mean	± 1σ	Median	Min	Max
Afrosoricida		18; ♂ = 13; ♀ = 5	−23.2	0.5	−23.1 ^1^	−24.2	−22.4	7.1	0.9	7.1 ^1^	5.8	8.5
	*Hemicentetes semispinosus*	3; ♂ = 3	−23.1	0.6	−23.1 ^a^	−23.6	−22.5	6.1	0.1	6.1 ^a^	6.0	6.2
	*Nesogale dobsoni*	2; ♂ = 2	−23.0	0.2	−23 ^a^	−23.1	−22.8	6.3	0.6	6.3 ^a^	5.8	6.7
	*Nesogale talazaci*	1; ♀ = 1	−23.6		−23.6 ^a^	−23.6	−23.6	5.9		5.9 ^a^	5.9	5.9
	*Oryzorictes hova*	9; ♂ = 5; ♀ = 4	−22.9	0.4	−22.8 ^a^	−23.7	−22.4	0.7	0.8	8.0 ^a^	6.6	8.5
	*Setifer setosus*	3; ♂ = 3	−23.9	0.4	−24.1 ^a^	−24.2	−23.5	7.5	0.4	7.5 ^a^	7.2	7.9
		Species comparison:			χ^2^ = 7.16, df = 4, *p* = 0.13					χ^2^ = 10.45, df = 4, ***p* = 0.033**		
					Levene *p* = 0.42					Levene *p* = 0.13		
		Excluding *Neogale talazaci*:			χ^2^ = 5.21, df = 3, *p* = 0.16					χ^2^ = 5.21, df = 3, ***p* = 0.030**		
					Levene *p* = 0.74					Levene ***p* = 0.049**		
Chiroptera		27; ♂ = 19; ♀ = 8	−23.8	0.9	−24.2 ^2^	−24.7	−21.0	7.2	0.9	7.2 ^1^	4.8	8.8
	*Macronycteris commersoni*	1; ♀ = 1	−21.9		−21.9 ^ab^	−21.9	−21.9	8.0		8.0 ^ab^	8.0	8.0
	*Mops leucostigma*	6; ♂ = 3; ♀ = 3	−22.6	0.8	−22.9 ^a^	−23.3	−21.0	8.3	0.5	8.5 ^a^	7.5	8.8
	*Myotis goudoti*	10; ♂ = 6; ♀ = 4	−24.2	0.2	−24.2 ^b^	−24.5	−23.9	7.0	0.3	7.0 ^b^	6.4	7.3
	*Myzopoda aurita*	9; ♂ = 9	−24.3	0.3	−24.4 ^b^	−24.7	−23.6	7.1	0.7	7.1 ^ab^	6.2	8.1
	*Rousettus madagascariensis*	1; ♂ = 1	−23.5		−23.5 ^ab^	−23.5	−23.5	4.8		4.8 ^ab^	4.8	4.8
		Species comparison:			χ^2^ = 18.05, df = 4, ***p* = 0.0012**					χ^2^ = 14.67, df = 4, ***p* = 0.0054**		
					Levene ***p* = 0.020**					Levene ***p* = 0.043**		
		Excluding *Macronycteris commersoni* and *Rousettus madagascariensis*:			χ^2^ = 14.84, df = 2, ***p* = 0.0006**					χ^2^ = 11.48, df = 2, ***p* = 0.0032**		
					Levene ***p* = 0.020**					Levene ***p* = 0.043**		
		Excluding *Mops leucostigma*:			χ^2^ = 7.50, df = 3, *p* = 0.058					χ^2^ = 4.77, df =3, *p* = 0.19		
					Levene *p* = 0.68					Levene ***p* = 0.016**		
Eulipotyphla	*Suncus murinus*	1; ♂ = 1	−23.7		−23.7 ^123^	−23.7	−23.7	8.0		8.0 ^12^	8.0	8.0
Rodentia		14; ♂ = 6; ♀ = 8	−24.9	0.4	−24.9 ^3^	−25.7	−24.1	2.1	1.7	2.0 ^2^	−0.5	4.6
	*Eliurus minor*	5; ♂ = 2; ♀ = 3	−24.9	0.6	−24.7 ^a^	−25.7	−24.3	3.3	1.3	3.5 ^a^	1.9	4.6
	*Eliurus petteri*	1; ♀ = 1	−25.0		−25.0 ^a^	−25.0	−25.0	2.4		2.4 ^a^	2.4	2.4
	*Eliurus webbi*	5; ♂ = 2; ♀ = 3	−24.9	0.3	−2.05 ^a^	−25.3	−24.5	1.9	1.7	1.4 ^a^	−0.2	4.3
	*Rattus rattus*	3; ♂ = 2; ♀ = 1	−24.7	0.6	−24.7 ^a^	−25.3	−24.1	0.2	0.6	0.5 ^a^	−0.5	0.5
		Species comparison:			χ^2^ = 0.40, df = 3, *p* = 0.94					χ^2^ = 6.98, df = 3, *p* = 0.073		
					Levene *p* = 0.53					Levene *p* = 0.25		
		Excluding *Eliurus petteri*:			χ^2^ = 0,32 df = 2, *p* = 0.85					χ^2^ = 4.77, df =3, ***p* = 0.034**		
					Levene *p* = 0.53					Levene *p* = 0.25		
Order comparisons:					χ^2^ = 32.83, df = 3, ***p* < 0.0001**					χ^2^ = 32.37, df = 3, ***p* < 0.0001**		
					Levene ***p* = 0.034**					Levene ***p* = 0.0042**		
		Excluding *Suncus murinus*:			χ^2^ = 32.76, df = 2, ***p* < 0.001**					χ^2^ = 4.77, df =3, ***p* < 0.0001**		
					Levene ***p* = 0.022**					Levene ***p* = 0.0042**		
		Excluding *Mops leucostigma*:			χ^2^ = 36.60, df = 3, ***p* < 0.0001**					χ^2^ = 31.46, df = 3, ***p* < 0.0001**		
					Levene *p* = 0.82					Levene ***p* = 0.0006**		

**Table 4:** Summary isotopic data and statistical comparisons for small mammals from Betampona Réserve Naturelle Intégrale (BRNI) that were trapped in different regions (forest interior >900 m from the BRI boundary, forest edge <400 m from boundary, and outside of BRI), and (2) habitats (undisturbed moist forest, slightly degraded forest, second-growth forest, or anthropogenic village or agricultural areas). Significant results are presented in bold.

Order			δ^13^C (‰)			δ^15^N (‰)	
		N	Mean ± 1σ	Median	N	Mean ± 1σ	Median
Region comparisons							
Afrosoricida	Forest interior	18	−23.1 ± 0.5	−23.1	18	7.2 ± 0.9	7.1
Chiroptera	Forest interior	4	−24.3 ± 0.3	−24.3 ^b^	4	6.9 ± 0.3	7.1 ^b^
	Forest edge	17	−24.1 ± 0.7	−24.2 ^b^	17	6.9 ± 0.8	6.9 ^b^
	Outside of forest	6	−24.3 ± 0.3	−22.9 ^a^	6	8.3 ± 0.5	8.5 ^a^
				χ^2^ = 11.67, df = 2, ***p* = 0.0029** *			χ^2^ = 11.16, df = 2, ***p* = 0.0038** *
				Levene *p* = 0.38			Levene *p* = 0.35
Eulipotyphla	Forest interior	1	−23.7	−23.7	1	8.0	8.0
Rodentia	Forest interior	11	−25.0 ± 0.4	−25.1	11	2.3 ± 1.6	2.0
	Forest edge	3	−24.5 ± 0.3	−24.7	3	1.2 ± 2.1	0.5
				χ^2^ = 2.23, df = 1, *p* = 0.14			χ^2^ = 1.19, df = 1, *p* = 0.27
				Levene *p* = 0.66			Levene *p* = 0.63
Habitat comparisons							
Afrosoricida	Undisturbed moist forest	10	−23.2 ± 0.5	−23.1	10	7.2 ± 1.0	7.5
	Slightly degraded moist forest	8	−23.0 ± 0.5	−23.0	8	7.1 ± 1.0	6.9
				χ^2^ = 0.80, df = 1, *p* = 0.37			χ^2^ = 0.071, df = 1, *p* = 0.79
				Levene *p* = 0.97			Levene *p* = 0.76
Chiroptera	Slightly degraded moist forest	4	−24.3 ± 0.3	−24.3 ^b^	4	6.9 ± 0.3	7.1 ^b^
	Second-growth forest	17	−24.1 ± 0.7	−24.2 ^b^	17	6.9 ± 0.8	6.9 ^b^
	Anthropogenic habitat	6	−24.3 ± 0.3	−22.9 ^a^	6	8.3 ± 0.5	8.5 ^a^
				χ^2^ = 11.67, df = 2, ***p* = 0.0029** *			χ^2^ = 11.16, df = 2, ***p* = 0.0038** *
				Levene *p* = 0.38			Levene *p* = 0.35
Eulipotyphla	Slightly degraded moist forest	1	−23.7	−23.7	1	8.0	8.0
Rodentia	Undisturbed moist forest	5	−25.1 ± 0.4	−25.1	5	3.5 ± 1.4	4.3
	Slightly degraded moist forest	9	−24.7 ± 0.4	−24.7	9	1.3 ± 1.3	1.4
				χ^2^ = 2.61, df = 1, *p* = 0.11			χ^2^ = 5.16, df = 1, ***p* = 0.023**
				Levene *p* = 0.50			Levene *p* = 0.85

* For bats, groups that share a superscript letter are statistically indistinguishable based on Steel–Dwass All Pairs post hoc tests.

## Data Availability

All data generated for this project are provided in Supplementary Material Tables S1 and S2. They have also been added to the publicly available IsoMad database associated with [12].

## Supplementary Materials

The following supporting information can be downloaded at https://www.mdpi.com/article/10.3390/ani15162423/s1, Table S1: Taxonomic, elemental, isotopic, and data for individual plant samples collected at Betampona Réserve Naturelle Intégrale, Table S2: Details for individual small mammal specimens, including raw isotope data, Table S3: Summary isotope data for fur keratin, raw bone collagen, and converted collagen for each species, and Table S4: Comparison of isotope data for small mammal taxa for the two sample periods (2015 and 2016).
